# Activation of VIPR1 suppresses hepatocellular carcinoma progression by regulating arginine and pyrimidine metabolism

**DOI:** 10.7150/ijbs.71134

**Published:** 2022-07-04

**Authors:** Yaojie Fu, Shanshan Liu, Robim M. Rodrigues, Ying Han, Cao Guo, Zhanwei Zhu, Yong He, Bryan Mackowiak, Dechun Feng, Bin Gao, Shan Zeng, Hong Shen

**Affiliations:** 1 Department of Oncology, Xiangya Hospital, Central South University, Changsha, 410008, Hunan, China; 2 Laboratory of Liver Diseases, National Institute on Alcohol Abuse and Alcoholism, National Institutes of Health, Bethesda, MD, 20892, USA; 3 National Clinical Research Center for Geriatric Disorders, Xiangya Hospital, Central South University, Changsha, 410008, Hunan, China

**Keywords:** arginine metabolism, pyrimidine synthesis, VIPR1, ASS1, CAD

## Abstract

**Background and aims:** Vasoactive intestinal polypeptide type-I receptor (VIPR1) overexpression has been reported in numerous types of malignancies and utilized to develop novel target therapeutics and radiolabeled VIP analogue-based tumor imaging technology, but its role in liver carcinogenesis has not been explored. In the current study, we investigated the role of the VIP/VIPR1 signaling in controlling hepatocellular carcinoma (HCC) progression.

**Approach and results:** By analyzing clinical samples, we found the expression level of VIPR1 was downregulated in human HCC tissues, which was correlated with advanced clinical stages, tumor growth, recurrence, and poor outcomes of HCC clinically. *In vitro* and in *vivo* studies revealed that activation of VIPR1 by VIP markedly inhibited HCC growth and metastasis. Intriguingly, transcriptome sequencing analyses revealed that activation of VIPR1 by VIP regulated arginine biosynthesis. Mechanistical studies in cultured HCC cells demonstrated that VIP treatment partially restored the expression of arginine anabolic key enzyme argininosuccinate synthase (ASS1), and to some extent, inhibited *de novo* pyrimidine synthetic pathway by downregulating the activation of CAD (carbamoyl-phosphate synthetase 2, aspartate transcarbamylase, and dihydroorotase). VIP treatment upregulated ASS1 and subsequently suppressed CAD phosphorylation in an mTOR/p70S6K signaling dependent manner. Clinically, we found human HCC samples were associated with downregulation of ASS1 but upregulation of CAD phosphorylation, and that VIPR1 levels positively correlated with ASS1 levels and serum levels of urea, the end product of the urea cycle and arginine metabolism in HCC.

**Conclusions:** Loss of VIPR1 expression in HCC facilitates CAD phosphorylation and tumor progression, and restoration of VIPR1 and treatment with the VIPR1 agonist may be a promising approach for HCC treatment.

## Introduction

Hepatocellular carcinoma (HCC) is the most common primary liver malignancy and the second cause of cancer-related death globally 1. Despite some achievements in the management of HCC, most HCC patients diagnosed with advanced HCC stages have poor clinical outcomes 2. Hence, it is urgent to develop more predictive biomarkers and novel therapeutic approaches to improve the survival rate of HCC patients.

Vasoactive intestinal peptide (VIP) is a 28-amino acid neurotransmitter that plays a critical role in regulating a wide variety of pathophysiological functions, such as neuroprotection, vasorelaxant effects, and the control of innate and adaptive immune responses 3. In the liver, VIP plays an important role in maintaining homeostasis of several physiological processes. Recent single cell RNA sequencing (scRNA-seq) analyses indicated VIP mainly originates from endocrine and/or neuroendocrine organs 4. It has been reported that VIP induces bile transport and secretion by stimulating cellular activities in both biliary epithelial cells and hepatocytes 5, and that periportal mesenchymal cell-derived VIP participates in tight junctions and iron/water transporter establishment in bile duct development 6. Additionally, several studies suggest that the VIP-mediated cAMP-PKA signaling exerts hepatocellular protection and diminishes inflammation by inhibiting neutrophil and macrophage infiltration during hepatic ischemia/reperfusion injury (IRI) 7, 8. However, the role of VIP in liver carcinogenesis remains obscure.

In most cases, VIP exerts its functions by binding two types of receptors, type 1 and 2 VIP receptors (VIPR1, VIPR2), and the liver mainly expresses VIPR1 9, 10. VIPR1, a member of the G protein-coupled receptors (GPCRs) expressed on the cell membrane, has a wide distribution in both central nervous system (CNS) and peripheral organs 11. VIP/VIPR1 signaling has been implicated in the pathogeneses of various types of human diseases, such as metabolic diseases, neurological disorders, and autoimmune diseases 10, 11. Recently, the significance of VIP/VIPR1 signaling in carcinogenesis came to light 12. VIPR1 was found to be frequently overexpressed in a range of malignancies, such as gastric cancer, lung cancer, renal cancer, and breast cancer 13-16. However, the characteristics of VIPR1 and VIP/VIPR1 signaling in HCC remain to be determined.

In the current study, we investigated the association between VIPR1 and HCC clinically by examining VIPR1 expression in HCC and non-tumorous liver tissues. Our data revealed that VIPR1 expression was significantly decreased in HCC, and downregulation of VIPR1 was strongly related to HCC proliferation, progression, and poor clinical outcomes. We further demonstrated that activation of VIPR1 by VIP plays a critical role in hindering HCC growth and metastasis *in vitro* and* in vivo*. Moreover, the RNA-seq data indicated that VIP/VIPR1 signaling upregulates arginine anabolic enzymes expression and inhibits carbamoyl-phosphate synthetase 2, aspartate transcarbamylase, and dihydroorotase (CAD)-mediated pyrimidine biosynthesis, suggesting its involvement in metabolic regulation of HCC. Mechanistically, we found that HCC development was closely associated with decreased ASS1 expression and upregulated phosphorylated CAD (p-CAD). Activation of VIP/VIPR1 signaling elevated ASS1 level, which resulted in the downregulation of CAD phosphorylation by inhibiting mTOR/p70S6K signaling pathway. Finally, in HCC patients, we determined the potential of VIPR1, p-CAD, and ASS1 to serve as predicting biomarkers in HCC recurrence, which may provide novel biomarkers and new therapeutic approaches for HCC treatment.

## Materials and Methods

### Human HCC samples

Human plasma, hepatocellular carcinoma (HCC) tissues, and adjacent non-tumor liver tissues were obtained from HCC patients who had undergone surgical resection in Xiangya Hospital from 2005 to 2019. The studies involving human samples were reviewed and approved by Xiangya Hospital Ethics Committee. The collection, utilization and analysis of all clinical samples were in accordance with institutional requirements. Information for the characteristics of each patient in all clinical cohorts recruited in the manuscript is included in Supplementary Tables 1-6.

### Mouse model

For the development of the subcutaneous cell derived xenograft (CDX) model, 5×10^6^ HCCLM3 cells resuspended in 50-100 μl 1×PBS were injected subcutaneously into the dorsal region of 4 to 5 week old male athymic nude mice (Nanjing Biomedical Research Institute of Nanjing University), followed by VIP (i.v. 300µg/kg) (Tocris Minneapolis, MN) or placebo treatment. For the HCC metastatic model, 1×10^6^ HCCLM3 cells resuspended in 50μl 1×PBS were injected to NOD-*Prkdc*^em26Cd52^*Il2rg*^em26Cd22^/*Nju* (NOD/SCID *IL2rg*^-/-^) mice (shorten as NCG mice; Nanjing Biomedical Research Institute of Nanjing University) via tail vein. All animal procedures on CDX model were conducted in the Central South University in accordance with protocols approved by IACUC of Central South University.

The diethylnitrosamine (DEN)-induced liver cancer model was induced by intraperitoneally injecting male C57BL/6J mice with DEN (Sigma) (25mg/kg in PBS) at 15 days after birth. The mice were sacrificed 9 months after DEN injection. All animal procedures on DEN model were conducted at the NIAAA in accordance with protocols approved by IACUC of the NIAAA.

### Bioinformatic analysis on TCGA cohort

Bioinformatic analyses were performed based on transcriptomic data from publicly available datasets of HCC cohorts, including The Cancer Genome Atlas (TCGA, http://cancergenome.nih.gov/) and UCSC Xena (https://xena.ucsc.edu/). *VIPR1* expression and clinical information, including Overall survival (OS), Relapse-free survival (RFS), histologic grade, and pathologic parameters of TCGA hepatocellular carcinoma cohort (TCGA-LIHC cohort) were used. The related statistical analysis was performed using R software (3.2.2). All data acquirement and application were in accordance with TCGA publication guidelines and policies.

### Statistical analysis

Data are presented as the mean±SEM and were analyzed using GraphPad Prism software (v. 5.0a; GraphPad Software, La Jolla, CA). Logistic regression analyses to calculate the odds ratio (ORs) and 95% confidence interval (95% CI) were performed. Significance was evaluated by Student's *t*-test for comparison of two groups or by one-way analysis of variance (ANOVA) for comparison of multiple groups. P values of <0.05 were considered to be significant.

All other materials and methods are included in the Supporting materials.

## Results

### Downregulated expression of VIPR1 in HCC correlates with enhanced HCC proliferation, clinical stages and poor prognosis

**Figure 1:**
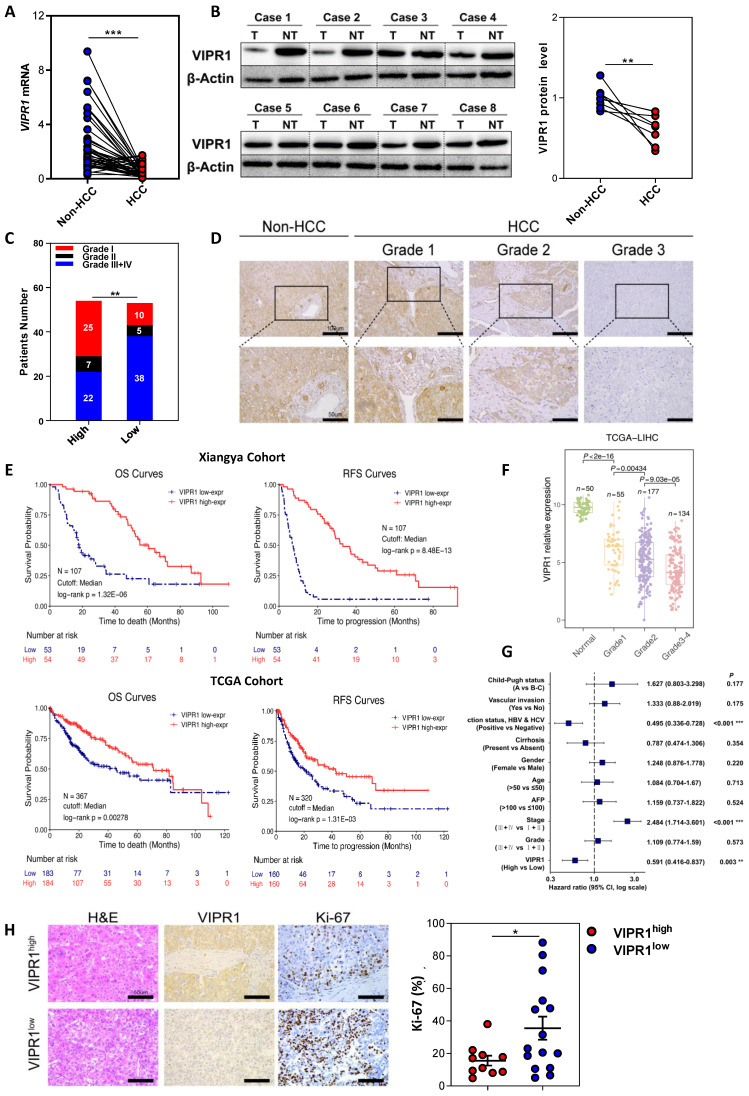
** Loss of VIPR1 in HCC is associated with tumor aggressiveness and poor prognosis clinically. (A)** *VIPR1* mRNA levels in paired HCC samples and adjacent non-tumor liver (n=41). **(B)** VIPR1 protein levels in paired tissues (n=8). **(C)** Clinical staging composition in *VIPR1*^high^ (n=54) and *VIPR1*^low^ groups (n=53). **(D)** Representative immunohistochemical (IHC) staining of VIPR1 in non-tumor liver and Grade 1-3 HCC tissues. Squared area in the top lane is shown in higher magnification in lower panels. **(E)** Kaplan-Meier curves comparing the overall survival (OS) and relapse-free survival (RFS) in HCC patients with low- and high- *VIPR1* expression in Xiangya cohort (n=107; log-rank test; cutoff value: median) and TCGA-LIHC cohort (n=320; log-rank test; cutoff value: median). **(F)** *VIPR1* gene expression level comparison among normal liver (shown as Normal) and Grade 1-4 HCC tissues. **(G)** Multivariable Cox regression analysis on TCGA-LIHC cohort. **(H)** Representative H&E, Ki-67 staining, and Ki-67 index analysis in VIPR1^high^ (n=10) and VIPR1^low^ (n=15) HCC tissues. Two-tailed paired Student's *t* test with Welch's correction was performed in panels A and B; unpaired Student's *t* test was performed in panels F and H. Values represent means±SEM. *P< 0.05, **P< 0.01, ***P< 0.001.

To investigate the role of VIPR1 in regulating HCC progression, we first examined *VIPR1* mRNA levels in 41 paired HCC and adjacent non-tumor tissues. In another independent cohort (n=8), VIPR1 protein levels were validated. Our data showed that both mRNA and protein levels of VIPR1 were significantly downregulated in HCC tissues compared to the matched adjacent-non tumor tissues (Fig. 1A-1B). Moreover, downregulation of *Vipr1* mRNA and VIPR1 protein in HCC was also observed in a mouse model of diethylnitrosamine (DEN) -induced liver cancer (Fig. S1A-S1D).

To further analyze the potential effect of VIPR1 on tumor pathological characteristics, we performed univariable analysis in an HCC cohort of 107 post-surgical-patients to determine the differences between high- and low- *VIPR1*-expressed patient subgroups (Table 1). Our data revealed that *VIPR1* mRNA expression was inversely correlated with aggressive features of HCC, including the size and number of tumor mass, vascular invasion, distant metastasis, and advanced TNM stage (Table 1; Fig. 1C). Additionally, patients with relatively low VIPR1 expression exhibited worse pathological differentiated status (Fig. 1D). To further determine the association between *VIPR1* expression and clinical prognosis of HCC patients, we performed Kaplan-Meier survival analysis of the cohort and found that patients with low *VIPR1* expression had worse overall survival (OS) and relapse-free survival (RFS) than those with high *VIPR1* expression (Fig. 1E). A similar conclusion was also obtained from Kaplan-Meier survival analysis of The Cancer Genome Atlas Liver Hepatocellular Carcinoma (TCGA-LIHC) cohort (Fig. 1E). Furthermore, analysis of TCGA-LIHC cohort also revealed that *VIPR1* expression was significantly downregulated in HCC tissues compared to normal liver tissues (Fig. S1E), and its expression levels negatively correlated with tumor grade (Fig. 1F). In addition, by using multivariable Cox regression analysis, we found that advanced clinical stage was a hazard factor for tumor progression; however, *VIPR1* mRNA expression level represented an independent predictor for better prognosis of HCC (Fig. 1G).

To assess the role of VIPR1 in HCC proliferation, we performed Ki-67 IHC staining and found that HCC tissues with low VIPR1 expression were characterized by higher a Ki-67 index (Fig. 1H). Moreover, bioinformatic analysis of the TCGA-LIHC cohort also showed a remarkably negative correlation between *VIPR1* and *MKI67* or Proliferating Cell Nuclear Antigen (*PCNA*) expression (Fig. S1F).

Finally, we performed a prospective study of a post-surgery recurrent cohort. The waterfall plot analysis indicated that *VIPR1* expression was inversely associated with HCC relapse (Fig. S1G). Similarly, spider plot analysis showed *VIPR1* levels negatively correlated with the relapsed tumor size after surgery (Fig. S1H).

### VIPR1 agonist VIP inhibits HCC proliferation and migration *in vitro*

**Figure 2:**
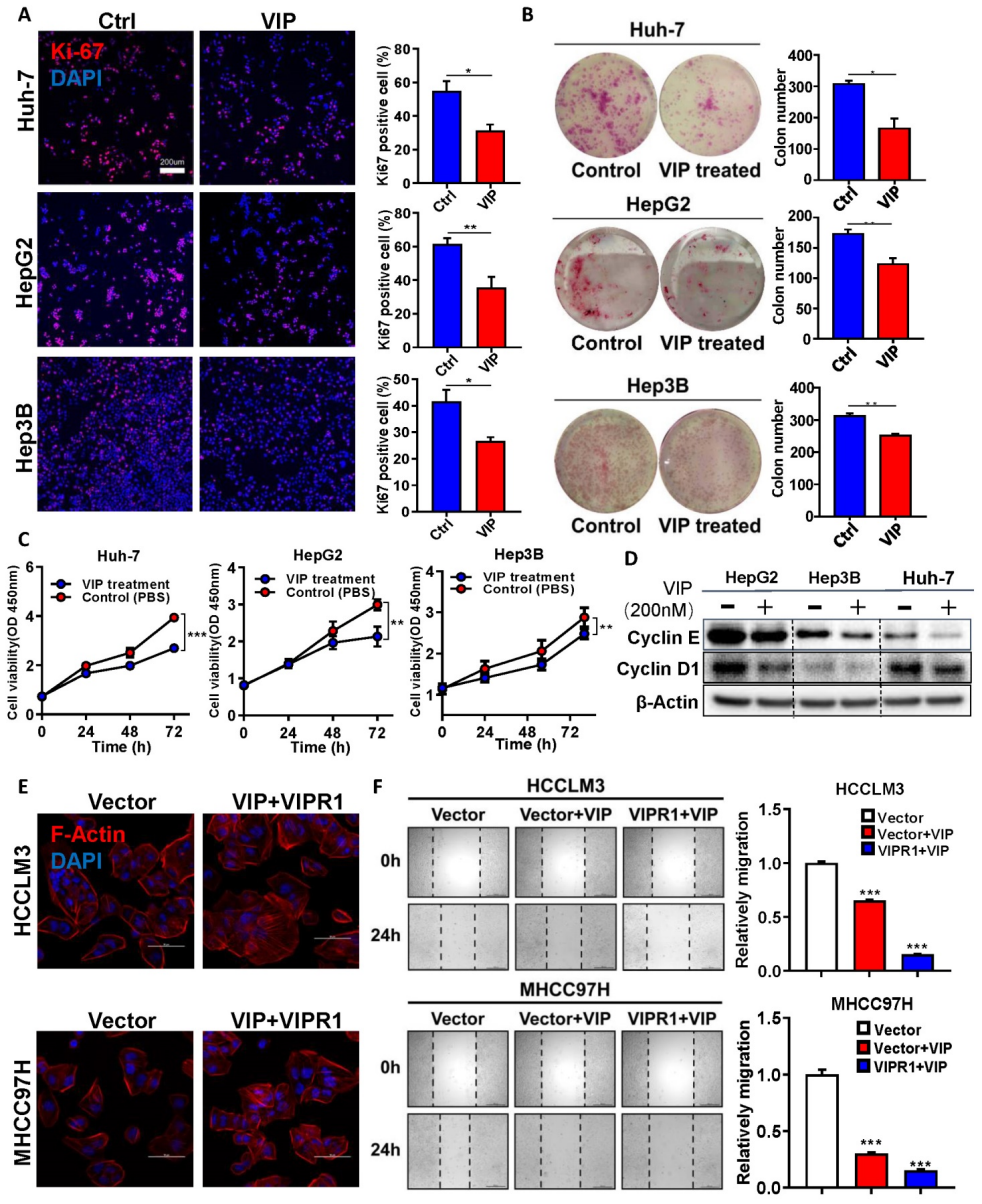
** Activation of VIPR1 by VIP inhibits HCC cell growth and migration *in vitro*. (A)** Ki-67 immunofluorescence staining of Control (Ctrl) and VIP (200nM) treated HCC cell lines (HepG2, Hep3B and Huh-7) (scale bars, 200μm). **(B)** Representative images of colony formation in Ctrl and VIP treated groups (Hep3B and Huh-7 cells were seeded at 1000 cells per well; HepG2 cell were seeded at 5000 cells per well. The cells were kept in culture for15 days). **(C)** Cell proliferative capacity was monitored by CCK-8 assay for about 72h. **(D)** Western Blot analysis of cyclin E and cyclin D1 in Ctrl and VIP treated cells. **(E)** Phalloidin staining for cytoskeletal structure in Vector-HCCLM3 cell and Vector-MHCC97H cell (shown as Vector) and VIP treated lenti-VIPR1 overexpressing HCCLM3 cell and MHCC97H cell (namely the VIP/VIPR1 signaling activation group, shown as VIP+VIPR1). Blue: DAPI; Red: F-Actin. **(F)** Wound healing assay of placebo (PBS) treated Vector-HCCLM3 (shown as Vector); VIP treated lenti-VIPR1 overexpressing HCCLM3 cell (shown as VIPR1+VIP), VIP treated Vector-HCCLM3 cell (shown as Vector+VIP). All groups were treated for 24h *in vitro*. Two-paired unpaired Student's *t* test was performed in (A). Values represent means±SEM. *P< 0.05, **P< 0.01, ***P< 0.001.

To further define the functional significance of VIPR1 in HCC, we examined *VIPR1* mRNA levels in the human hepatocyte cell line (LO2) and seven human HCC cell lines by performing RT-qPCR analysis (Fig. S2A), and identified three relatively *VIPR1* high-expression cell lines, namely Hep3B, HepG2, and Huh-7. Then we treated these three cell lines with VIP, a well-known specific agonist of VIPR1, and found that VIP exposure markedly inhibited cell viability in a dose-dependent manner (Fig. S2B) and attenuated the proliferative capacity of the cells, as evidenced by the reduction of Ki-67 index, colony formation numbers, and cell proliferation (Fig. 2A-2C). Furthermore, VIP treatment downregulated the expression of several cell-cycle markers such as Cyclin E and Cyclin D1 in HCC cell lines (Fig. 2D). Consistently, VIP intervention also decreased the activation of several signaling pathways (eg. STAT3, AKT, and ERK1/2) that promote cellular survival and tumor growth (Fig. S2C).

Next we examined whether VIP may play a role in regulating HCC migration. To answer this question, we established stable VIPR1 overexpressing cell lines in HCCLM3 and MHCC97H cells in which VIRP1 expression is relatively low (Fig. S2A). A CCK8 assay demonstrated that treatment with VIP attenuated the growth of these cells (Fig. S2D). In addition, the morphological analyses revealed that treatment with VIP reduced broad lamellipodia, spike-like filopodia, and polymerization of actin filaments in HCCLM3 and MHCC97H cells with VIRP1 overexpression compared to those with vector expression, which partially contributed to the decreased migrating and metastatic capacity of tumor cell 17 (Fig. 2E; Fig. S2E). Consistent with morphological changes, the wound healing assay demonstrated that VIP intervention attenuated HCC migrating potential with greater inhibition in HCCLM3 cells with VIPR1 overexpression than those with vector expression (Fig. 2F).

### VIPR1 agonist VIP suppresses HCC growth and distant metastasis *in vivo*

**Figure 3:**
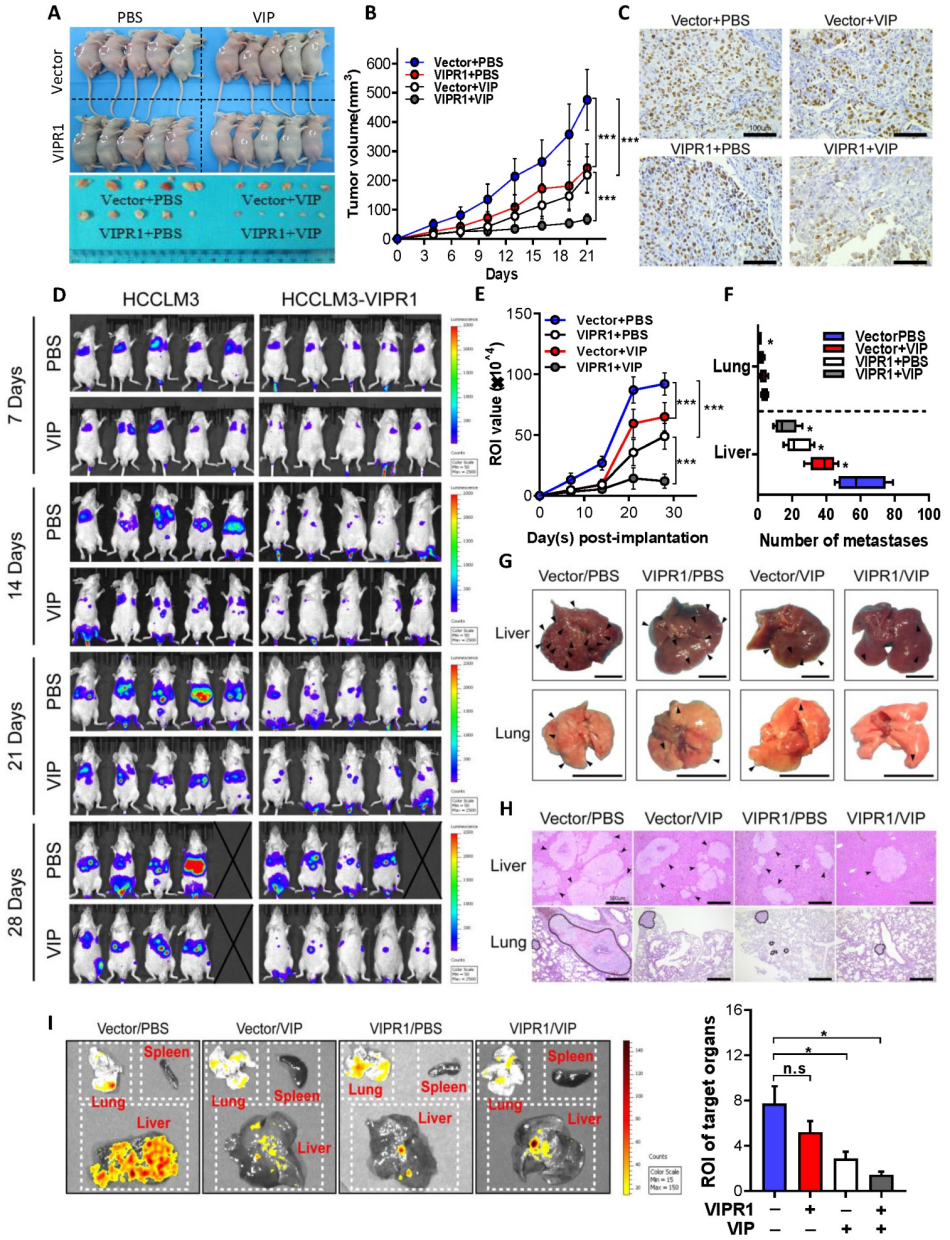
***In vivo* activation of VIPR1 by VIP treatment suppresses HCC growth and distant metastasis. (A-C)** HCCLM3-VIPR1 cells with VIRP3 overexpression and HCCLM3-Vector cells with vector expression were subcutaneously implanted in the backs of nude mice (5×10^6^ cells/mouse; n=5 in each group), followed by VIP (i.v., 300µg/kg) or vehicle treatment. The mice were sacrificed at Day 21. The volume of tumor masses is shown in panel A. Tumor size was measured twice per week in 3 continuous weeks and is shown in panel B. Representative Ki-67 staining of xenograft sections in each group (scale bars, 100 μm) is shown in panel C. **(D-I)**. Luciferase labeled Vector and HCCLM3-VIPR1 cells were intravenously injected to NCG mice (1×10^6^ cells/mouce; n=5 in each group), followed by VIP (i.p., 300µg/kg) or equal volume vehicle treatment. Bioluminescence imaging was subsequently used to visualize and quantify metastatic foci for 4 weeks and is shown in panel D. Photon flux of metastatic foci in Region of Interest (ROI) were quantified by recording bioluminescence value at week 1, 2, 3 and 4, and is shown in panel E. Number of metastatic foci in liver and lung were examined and counted at week 4 after sacrificing the mice and is shown in panel F. Metastases in targeted organs are indicated by arrows (scale bars, 1cm) and shown in panel G. Representative H&E staining of liver and lung sections in each group (scale bars, 500μm) is shown in panel H (metastases were surrounded by dotted line). Quantification of metastases in spleen, lung, and liver (panel I). Two-way ANOVA was performed in panels B and E. Abbreviations: n.s.: not significant; ROI: Region of Interest. Values represent means±SEM. *P< 0.05, **P< 0.01, ***P< 0.001.

To investigate whether VIP can elicit anti-tumor effects *in vivo*, we established HCC xenograft mouse models by subcutaneously implanting VIPR1 overexpressing HCCLM3 cells (HCCLM3-VIPR1) or vector transduced with HCCLM3 cells (HCCLM3-Vector) in the back of nude mice. These mice were treated with placebo (sterile PBS) or VIP twice per week, and tumor size was measured every three days (Fig. S3A). As shown in Fig. 3A-B, and Fig. S3B-S3C, tumors on mice with HCCLM3-VIPR1 implant grew much slower than the tumors on mice with HCCLM3-vector implant. Treatment with VIP further reduced tumor growth with much greater inhibition in mice with HCCLM3-VIPR1 implants than in those with HCCLM3-vector implant. Consistently, Ki-67 IHC staining assay revealed that VIP treatment markedly reduced HCC proliferation in the HCC xenograft mouse model with greater inhibition in mice with HCCLM3-VIPR1 implant than in mice with HCCLM3-vector implant (Fig. 3C).

Next, we determined whether treatment with VIP regulated tumor distant metastasis. HCCLM3-VIPR1 and HCCLM3-vector cells were transfected with luciferase and were injected into NCG mice for metastatic dissemination investigation. Mice were treated with VIP or placebo every other day, and subsequently subjected to *in vivo* bioluminescence imaging to monitor, visualize, and quantify fluorescent tracker labeled cancer metastases for 4 consecutive weeks (Fig. S3D). As illustrated in Fig. 3D, compared to the vector-expressed group, the VIPR1-overexpressed group had much less metastatic distribution in terms of all fluorescent foci. In addition, the number of metastatic foci towards liver and lung tissues was markedly reduced in the VIPR1-overexpressed group as well (Fig. 3E & 3F). Furthermore, histopathological and bioluminescence targeted organ detection analyses indicated that VIP treatment further restricted metastases formation in both groups (Fig. 3G-I), suggesting exogenous VIP treatment suppresses HCC metastasis via the activation of VIPR1.

### VIPR1 agonist VIP regulates arginine metabolism and pyrimidine biosynthesis in cultured HCC cells

**Figure 4:**
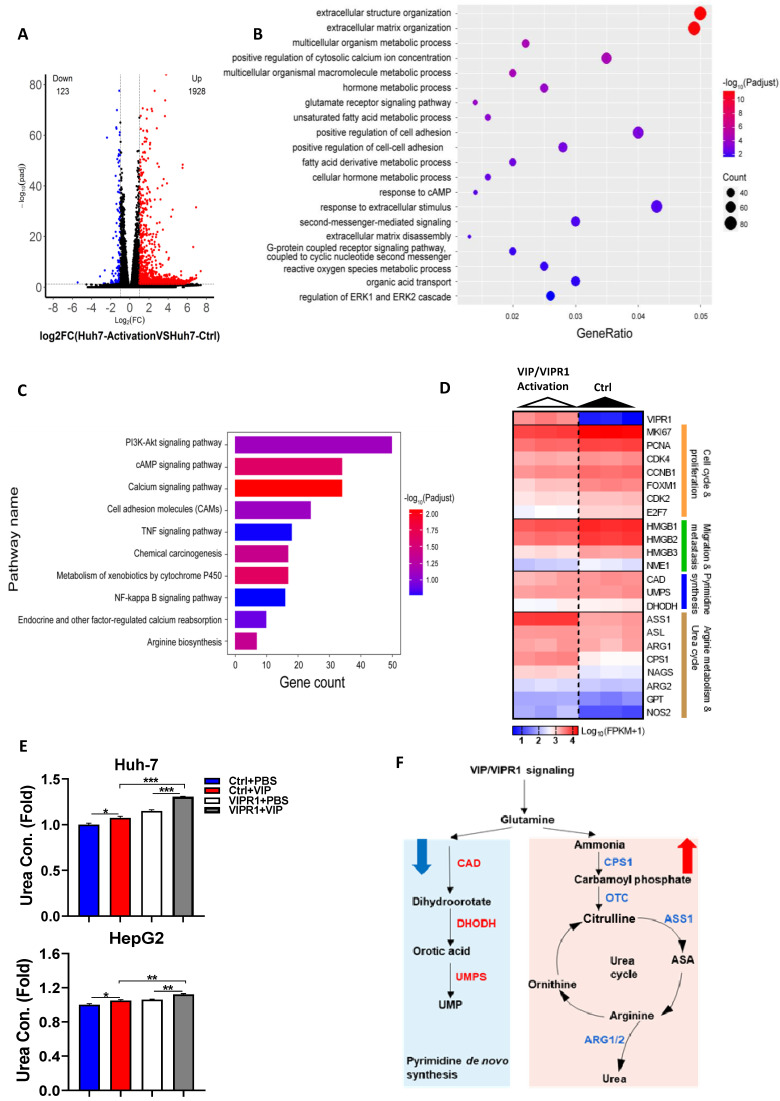
** A potential role of VIP/VIPR1 signaling in regulating arginine metabolism and pyrimidine biosynthesis of HCC.** Transcriptome sequencing analyses of Huh7 cell (Huh7-Ctrl), and VIP/VIPR1 axis activated Huh7 cell (Huh7-activation). **(A)** Volcano plot showed differentially expressed genes (DEGs) between two groups (1928 up-regulated genes; 123 down-regulated genes). **(B)** Gene Ontology (GO) enrichment analysis was performed on DEGs. **(C)** Kyoto Encyclopedia of Genes and Genomes (KEGG) enrichment analysis was performed on DEGs. **(D)** Heatmap showed increased expression of arginine synthesis and urea cycle genes (*ASS1, ASL, ARG1, ARG2, CPS1, NAGS, NOS2, GPT*), and lower expression of pyrimidine synthesis genes (*CAD, UMPS, DHODH*) in VIP/VIPR1 activation group. **(E)** Fold change of urea concentration in culture supernatant of different groups in HepG2 and Huh-7 cells. **(F)** Schematic diagram depicting a regulatory role of VIP/VIPR1 signaling in regulating arginine metabolism and pyrimidine biosynthesis of HCC. Abbreviations: Con.: concentration; n.s.: not significant. Values represent means±SEM. *P< 0.05, **P< 0.01.

To investigate the underlying mechanism by which VIP inhibits HCC progression, we performed RNA-seq analyses of Huh-7 cells with VIRP1 overexpression and VIP treatment (VIP/VIPR1 group) and Huh-7 cells with vector expression and without VIP treatment (control group) (Fig. S4A). Compared to the control group, the VIP/VIPR1 group had significant alterations of transcription profiles (Fig. S4A). Over 2000 differentially expressed genes (DEGs) were identified in the VIP/VIPR1 activation group *versus* control, showing that 1928 genes were upregulated, while 123 genes were downregulated (Fig. 4A). By utilizing Gene Ontology (GO) enrichment analysis, we found alterations of several gene sets that are related to inhibition of HCC in the VIP/VIPR1 group (Fig. 4B). To better understand the role of VIP/VIPR1 signaling in regulating HCC biological functions, we also used Kyoto Encyclopedia of Genes and Genomes (KEGG) enrichment to analyze transcriptome data and found that several tumorigenic pathways (such as PI3K-AKT, TNFα and NF-κB signaling pathways) were altered in the VIP/VIPR1 group *versus* the control group (Fig. 4C).

One of characteristics for tumor cells is to maximize their source of nitrogen by metabolic reprogramming to fuel their anabolic synthesis 18. For example, urea cycle dysregulation (UCD) is a common metabolic hallmark in various types of cancer, which is featured by downregulation of UC enzymes and several key enzymes in citrulline-arginine cycle, such as argininosuccinate synthase (ASS1), carbamoyl phosphate synthase 1 (CPS1), and ornithine carbamoyltransferase (OTC) 19, 20. In addition, UCD-associated arginine synthesis reduction elicits a 'metabolic shift', which recycles ammonia to glutamine and upregulates CAD activation to enhance *de novo* pyrimidine synthesis, promoting cancer initiation and progression 21. By analyzing transcriptome data, we found the expression of several metabolism related genes especially arginine biosynthesis-related genes were altered after VIP/VIPR1 activation (Fig. 4C). Treatment with the VIPR1 agonist VIP upregulated several arginine biosynthesis related genes (*ASS1, CPS1, ASL, ARG1, ARG2, NAGS, GPT, NOS2*) while the expression of *CAD* and *DHODH* were slightly downregulated in the VIP/VIPR1 cells *versus* control cells (Fig. 4D). Similarly, bioinformatic analysis of the TCGA-LIHC cohort also demonstrated that the expression of arginine biosynthesis related genes was relatively higher in *VIPR1*^high^ HCC patients (Fig. S4B). In agreement with the regulation of expression of these genes, treatment with VIP elevated levels of urea, the end product of the urea cycle and arginine metabolism, in cell culture supernatant of HepG2 and Huh7 cells. This elevation was more evident in the cells overexpressing VIPR1 than those transduced with the vector alone (Fig. 4E). Based on the above findings, we hypothesized that activation of VIPR1 by VIP may inhibit pyrimidine synthesis and arginine metabolism by downregulating CAD pathway and upregulating ASS1, respectively, which is summarized in Fig. 4E.

### VIP regulates arginine and pyrimidine metabolism by upregulating ASS1 that inhibits CAD phosphorylation in mTOR/p70S6K pathway dependent manner

**Figure 5:**
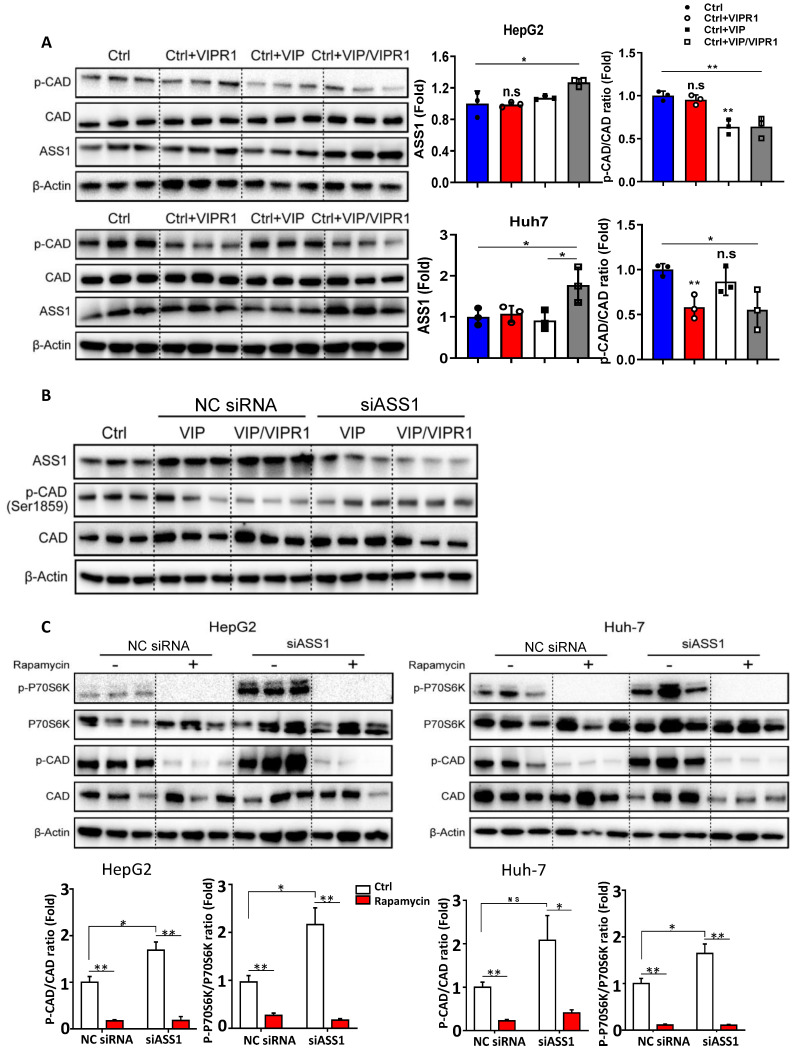
** Activation of VIPR1 by VIP decreases CAD phosphorylation by regulating ASS1 expression in an mTOR/p70S6K pathway dependent manner. (A)** HCC cell lines HepG2 and Huh7 were transfected with VIPR1 and treated with VIP *in vitro*, cellular extracts were subjected to Western blotting analyses. Independent experiments were conducted for three times. **(B)** VIP treatment alone (200nM for 48hrs; shown as VIP) or VIP/VIPR1 activation (200nM VIP treated VIPR1 plasmid transinfected cell for 48hrs; shown as VIP/VIPR1) in both negative control siRNA (NC siRNA) and siASS1 transinfected Huh-7 cells, then Western Blot was performed to measure protein level change of phosphorylated CAD (p-CAD) as compared to the control Huh-7 (Ctrl). (C) p-CAD/CAD ratio and p-p70S6K/ p70S6K ratio changes in NC siRNA and siASS1 transfected HepG2 and Huh-7 cells after rapamycin treatment *in vitro* (200nM; 2 hours). Independent experiments were conducted for three times. Unpaired Student's *t* test was performed in panels A and C. Values represent means±SEM. *P< 0.05, **P< 0.01.

To determine the molecular mechanisms by which VIP/VIPR1 regulates arginine and or pyrimidine synthesis in HCC cells, we overexpressed VIPR1 in HepG2 and Huh-7 cells via transfection and treated the cells with VIP (Fig. S5). As illustrated in Fig. 5A, overexpression of VIPR1 itself or the combination of VIPR1 and VIP decreased p-CAD levels in Huh-7 cells, while treatment with VIP or the combination of VIPR1 and VIP decreased p-CAD levels in HepG2 cells. In contrast, expression of ASS1 was markedly increased after treatment with VIP or the combination of VIPR1 and VIP.

Next, we sought to elucidate the mechanism by which VIP regulates phosphorylation of CAD in HCC. Based on previous studies 21, we hypothesized that ASS1 dysregulation caused by loss of VIPR1 in HCC may contribute to CAD activation. To test this notion, we first silenced *ASS1* by transfecting small interfering RNA (siASS1) in Huh-7 and HepG2 cells (Fig. S6A & S6B). We observed that cell proliferative capacity was increased after *ASS1* knockdown (Fig. S6C). In addition, knockdown of *ASS1* elevated p-CAD expression (Fig. S6B). Then, we transfected siASS1 in both control and VIPR1 overexpressed HCC cells, followed by treatment with VIP or vehicle control. As expected, levels of phosphorylated CAD were first reduced in VIP treatment alone or VIP/VIPR1 activated HCC cells and then restored by siASS1 transfection (Fig. 5B). Collectively, these results suggested that activation of VIPR1 by VIP increased ASS1 expression, which downregulated phosphorylation of CAD.

It has been reported that p70S6 kinase (p70S6K), a downstream target of mTOR signaling, can directly phosphorylate Ser1859 on CAD, which then catalyzes the first three reactions in pyrimidine *de novo* synthesis and stimulates cancer proliferation 21, 22. To investigate whether ASS1 controls CAD phosphorylation by regulating mTOR/p70S6K signaling, we utilized rapamycin (an mTOR inhibitor) to treat HepG2 and Huh-7 cells *in vitro.* The data revealed that rapamycin treatment resulted in decreased phosphorylation of CAD in both time- and dose-dependent manners (Fig. S6D-S6E). Moreover, we found that compared to the negative control siRNA (NC siRNA) transfected HCC cells, rapamycin treatment diminished the upregulation of both p-CAD and phosphorylated p70S6K (p-p70S6K) in the siASS1 group (Fig. 5C), suggesting ASS1 reduction upregulates CAD phosphorylation via the mTOR/p70S6K signaling pathway.

### Downregulation of ASS1 and upregulation of CAD phosphorylation in HCC correlate with VIPR1 levels and represent a potential biomarker for HCC recurrence

**Figure 6:**
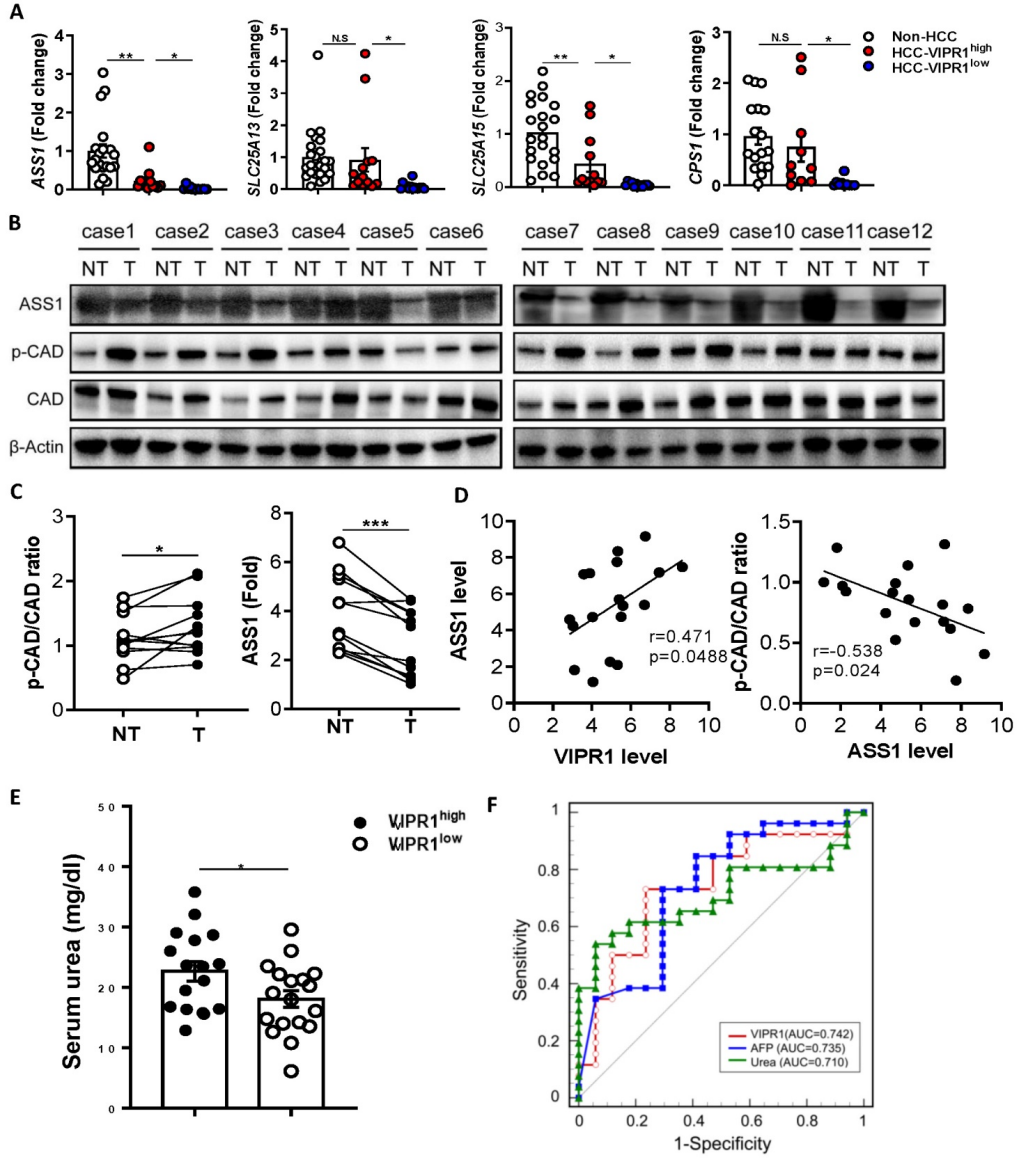
** Loss of VIPR1 correlates with ASS1 downregulation and increased phosphorylation of CAD. (A)** Expressions of arginine synthesis related genes (*ASS1, CPS1, SLC25A13, SLC25A15*) in human HCC and adjacent non-tumor samples were examined by qRT-PCR. **(B)** Protein levels of ASS1 and p-CAD/total CAD ratio in paired HCC and adjacent non-tumor clinical samples (n=12). **(C)** Quantification of ASS1 protein level, p-CAD/CAD ratio in paired HCC and non-tumor clinical samples (n=12). **(D)** Linear correlation between VIPR1 and ASS1 protein expression levels; and correlation between ASS1 and p-CAD/CAD ratio. **(E)** Preoperative serum urea level comparison between VIPR1^high^ (n=17) and VIPR1^low^ (n=18) HCC patients. **(F)** ROC curve of VIPR1 (AUC: 0.742; 95% CI: 0.586 to 0.863; p value: 0.0023); AFP (AUC: 0.735; 95% CI: 0.579 to 0.858; p value: 0.0038; cutoff value: 15.88ng/ml); Urea (AUC: 0.710; 95% CI: 0.552 to 0.838; p value:0.0077; cutoff value: 3.48mmol/l) in a prospective HCC cohort (n=43). Two-tailed paired Student's *t* test with Welch's correction was performed in panel C; Two-tailed p value and Pearson r value were showed in panel D; unpaired Student's *t* test was performed in panel E. Values represent means±SEM. *P< 0.05, **P< 0.01.

The above data suggest that VIP regulates arginine and pyrimidine metabolism by upregulating ASS1 that inhibits CAD phosphorylation in cultured HCC cells. Because CAD phosphorylation has been shown to promote carcinogenesis by enhancing pyrimidine *de novo* synthesis, while ASS1, a key regulator for urea cycle and arginine anabolic metabolism, has been shown to control carcinogenesis in several types of cancers 20, 21, 23-25, we wondered whether p-CAD and ASS1 are dysregulated in human HCC, and if such dysregulation is related to the loss of VIP/VIRP1. To test this notion, we performed RT-qPCR and western blot analyses of genes/proteins related to arginine metabolism and pyrimidine. RT-qPCR analyses of human HCC tissues revealed that levels of *ASS1, CPS1, SLC25A13, SLC25A15* were lower in HCC samples than in normal liver tissues with much lower levels in VIPR1^low^ HCC samples than in VIPR1^high^ HCC samples (Fig. 6A). Western blot analyses of ASS1 and phosphorylated CAD (p-CAD) in 12 paired clinical samples revelated that compared to non-tumor liver samples, ASS1 was notably downregulated, whereas CAD activation (p-CAD/CAD ratio) was upregulated in HCC tissues (Fig. 6B-C). The IHC staining further confirmed that ASS1 was highly expressed in parenchyma cells in non-tumor tissues but reduced in HCC tissues while p-CAD expression was significantly elevated in HCC tissues (Fig. S7A). Meanwhile, we validated our conclusion in DEN-induced HCC mouse model, which confirmed that HCC development correlated with phosphorylation of CAD and disrupted arginine anabolic metabolism (Fig. S7B-7C). Most importantly, we found a positive correlation between VIPR1 and ASS1 expression in 18 HCC samples, whereas a significant negative correlation was identified between ASS1 and p-CAD/CAD ratio (Fig. 6D).

The above *in vitro* and *in vivo* data suggest that VIP/VIPR1 is involved in regulating arginine metabolism in HCC. To further support this hypothesis, we measured serum urea, the end product of the urea cycle and arginine metabolism in HCC, and found that serum urea levels were much higher in HCC with VIPR1^high^ than those with VIPR1^low^ (Fig. 6E). Next, we evaluated the clinical significance of tumor VIPR1 and serum urea levels by measuring *VIPR1* expression of HCC tissue, serum urea, and AFP level in a cohort of 43 HCC patients. Our data revealed that VIPR1 and serum urea were two predictors for HCC relapse similar to AFP, as their area under curve (AUC) are comparable (Fig. 6F).

## Discussion

VIPR1 overexpression has been reported in numerous types of malignancies and has been utilized to develop novel target therapeutics and radiolabeled VIP analogue-based tumor imaging technologies 26, 27. Additionally, VIPR1 and VIP/VIPR1 signaling have received great attention in cancer research because of their pleiotropic roles in different types of cancer 28. For example, some studies indicated that VIP/VIPR1 signaling stimulates transactivation of HER2 and EGFR in human breast cancer. Small interference RNA (siRNA) directed VIPR1 silencing suppressed angiogenesis by inhibiting VIP effects on both VEGF secretion and EGFR/HER2 transactivation, which implied the use of VIPR1 antagonists for breast cancer treatment 29, 30. Additionally, recent reports suggest that VIP/VIPR1 signaling protects cancer stem cells (CSCs) from drug-induced apoptosis by dephosphorylating pro-apoptotic Bcl2 family member BAD 31.

**Figure 7:**
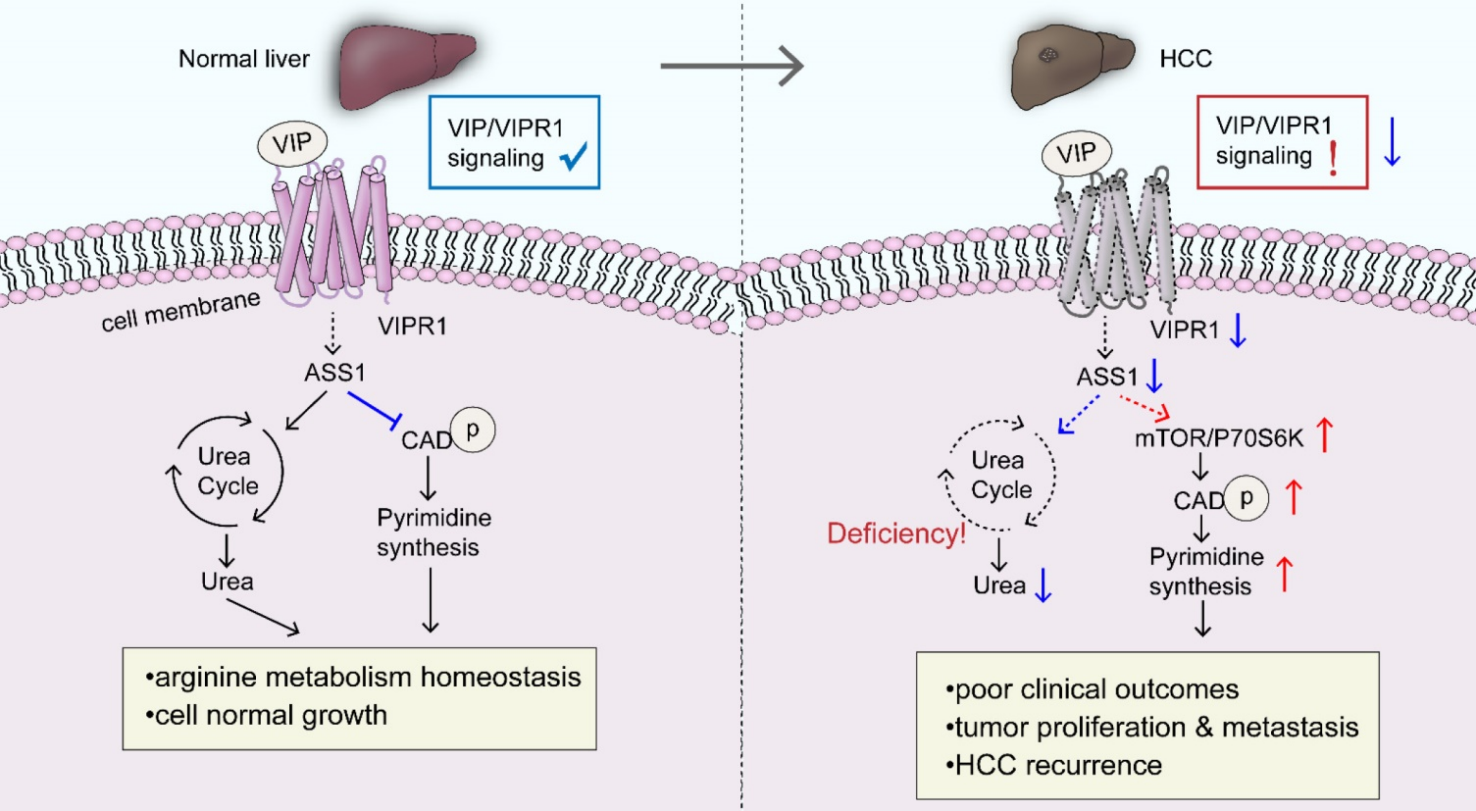
** A schematic overview depicting the potential role of VIP/VIPR1 signaling in controlling HCC development.** In normal physiological conditions, VIP/VIPR1 signaling plays a key role in maintaining arginine metabolic homeostasis and growth of the hepatocyte. The ligand VIP binds its Type-1 receptor, VIPR1, then mediates transmembrane signaling transduction. VIP/VIPR1 signaling modulates UC by regulating ASS1 level; additionally, ASS1 may partially participate in regulating pyrimidine synthesis by limiting phosphorylation of CAD, which contributes to normal cell growth and metabolic homeostasis. In contrast, during HCC, VIP/VIPR1 signaling is significantly downregulated due to the decrease/loss of VIPR1, which may be involved in ASS1 reduction. Moreover, ASS1 downregulation caused by inactivation of VIP/VIPR1 signaling can in turn promote HCC proliferation/recurrence through phosphorylation of CAD in an mTOR/p70S6K pathway-dependent manner. Abbreviations: UC: Urea cycle; CAD: Carbamoyl-Phosphate Synthetase 2; ASS1: argininosuccinate synthase.

More intriguingly, in gastric cancer, VIPR1/TRPV4/Ca^2+^ signaling stimulates VIP secretion, enforcing a positive feedback loop in promoting cancer progression 16. A similar growth stimulatory effect was observed in prostate cancer and neuroblastoma as well 32, 33. VIP/VIPR1 signaling maintained tumor proliferation and invasiveness by upregulating the expression of cyclins and angiogenic factors, including cyclin D1, MMP-2/MMP-9, VEGF, and COX-2, as well as stimulating tumor growth through the activation of PKA/ERK1/2 pathway 34. However, VIP/VIPR1 signaling has also been reported to inhibit tumor growth/proliferation. For instance, an early study revealed that VIP inhibits small cell lung cancer (SCLC) progression *in vitro* and *in vivo* via the activation of the cAMP/PKA pathway 35. In clear renal cell carcinoma (cRCC), some studies suggest that the proliferation-inhibitory role of VIP/VIPR1 signaling is related to the activation of EPAC/PI3K pathway 36. A similar inhibitory effect was also found in retinoblastoma and glioblastoma 37, 38. In the present study, we have demonstrated that VIPR1 is downregulated in HCC and that activation of VIPR1 by VIP inhibits HCC growth by regulating homeostasis of arginine metabolism and pyrimidine *de novo* synthesis, which are summarized in Fig. 7. An early investigation revealed* in vitro* treatment with VIP decreased activation of STAT3 and subsequently inhibited cell proliferation in HepG2 cells 39. Additionally, it was recently confirmed that VIP treatment increased HCC apoptosis by inhibiting the cAMP/Bcl-xL pathway 40. Our data revealed that loss of VIPR1 expression is associated with enhanced HCC growth, tumor grade and metastasis. More importantly, we found that downregulation of VIPR1 may serve as an independent predictor for HCC progression clinically. It would be interesting to explore what contributes to the reduction/loss of VIPR1 during hepatocarcinogenesis. Inspired by some related studies, we hypothesized there are two factors likely responsible for VIPR1 loss in HCC. First, it has been well defined that VIPR1 gene transcription is regulated by members of the nuclear receptor superfamily, including farnesoid X receptor (FXR) 9, a potent tumor suppressor of HCC that can directly repress oncogene transcription. Epigenetic silencing and inflammation are known to downregulate the expression of FXR in HCC during carcinogenesis 41, 42, which may be one possible reason for loss of VIPR1 in HCC. Second, some recent gene microarray analyses in HCC samples revealed that hypermethylation and H3K27 deacetylation in the promoter of VIPR1 results in its low expression 43, 44.

One interesting finding from our RNA-seq analyses is that VIP/VIPR1 signaling may be involved in maintaining HCC metabolic homeostasis, namely the urea cycle (UC), arginine biosynthesis, and pyrimidine* de novo* synthetic pathway. Under physiological conditions, UC takes place in the liver where UC enzymes regulate hepatic nitrogen disposal and provide amounts of intermediates for cellular metabolic need. Some arginine enzymes, such as ASS1, argininosuccinate lyase (ASL), and arginase (ARG), serve as distal UC enzymes responsible for urea generation and arginine synthesis, supplying the endogenous arginine for cell survival 18. However, multiple types of cancer, including HCC, exhibit aberrant enzyme expression and components in UC and arginine synthesis, which rewire the anabolism with reduced product of urea and increased redirection of carbon and nitrogen to pyrimidine synthesis 45. In the current study, we demonstrated for the first time that VIP/VIPR1 signaling regulated the expression of several arginine/UC key enzymes in HCC, such as *ASS1, CPS1, ASL,* and *ARG1/2.* Alterations of these genes may contribute to HCC progression by dysregulating arginine metabolism. In addition, increased phosphorylation of CAD has been observed in a variety of tumors, which stimulates tumor growth by initiating the first three reactions of pyrimidine *de novo* synthesis 20. Several reports indicated that ASS1 can be regulated by oncogene/tumor suppressors (such as MYC, p53) and epigenetic modifications 46, 47, and ASS1 expression is downregulated in HCC. However, little is known about the cancer-promoting mechanisms fostered by the reduction of ASS1. Although increasing evidence suggests that ASS1 deficiency drives metabolic adaptations to support tumor cell growth, in some cases, ASS1 contributes to cancer cell survival by modulating autophagy and signal transduction 48. These contradictory findings indcate the complex functions of ASS1. In the current study, we provided a cause-and-effect connection between downregulation of ASS1 and CAD activation in HCC. Our data revealed that decreased ASS1 caused by attenuation of VIP/VIPR1 signaling in HCC promotes phosphorylation of CAD through the mTOR/p70S6K pathway, which then promotes HCC growth/recurrence and proliferation.

*Clinical implications of the current study*: Our finding suggests that loss of VIPR1 expression promotes HCC aggressiveness, representing a novel regulatory effect during HCC development. Moreover, correlations between VIP/VIPR1 signaling and arginine metabolic flexibility provide a foundation for developing HCC innovative metabolic therapeutics and new prognostic biomarkers. There are several clinical implications of our study. First, besides the prognostic value of VIPR1 as mentioned above, serum urea can be exploited as a non-invasive indicator for prognosis and dynamic monitoring in HCC clinically. Second, arginine metabolic enzyme ASS1 and the pyrimidine synthetic pathway not only can serve as emerging biomarkers for post-surgery recurrence assessment, but can also represent a potential therapeutic option targeting the abnormal metabolism of HCC. Pegylated arginine deiminase, ADI-PEG20, has been utilized to treat ASS1 deficient and arginine auxotrophic tumors 49. Strikingly, initial phase I/II clinical trials with ADI-PEG20 showed partial responses and good tolerability in advanced HCC patients 50, which may promote clinical studies on targeting arginine metabolic vulnerability of HCC. Finally, VIP is used as a potent vasorelaxant drug clinically, so it will be interesting to test whether treatment with VIP or other VIPR1 agonists will generate beneficial effects for the treatment of HCC.

## Supplementary Material

Supplementary materials and methods, figures and tables.

## Figures and Tables

**Table 1:** Pathological characteristics of post-surgical HCC patients enrolled in Xiangya cohort

Variables		High VIPR1 Group (n=54)	Low VIPR1 Group(n=53)	P value
**Age (years)**		55.3±1.4	51.6±1.4	0.067
**Gender, n (%)**	Male	47 (87.0)	42 (79.2)	0.281
	Female	7 (13.0)	11 (20.8)	
**AFP, n(%)**	≥100ng/ml	25 (46.3)	30 (56.6)	0.286
	<100ng/ml	29 (53.7)	23 (43.4)	
**Child-Pugh classification**	A	21 (38.9)	16 (30.2)	0.344
	B+C	33 (61.1)	37 (69.8)	
**Tumor multiplicity, n (%)**	Solitary	37 (68.5)	25 (47.2)	*0.025
	Multiple	17 (31.5)	28 (52.8)	
**Tumor diameter, n (%)**	≥7cm	18 (33.3)	28 (52.8)	*0.042
	<7cm	36 (66.7)	25 (47.2)	
**Vascular invasion, n (%)**	Y	20 (37.0)	38 (71.7)	***0.0003
	N	34 (63.0)	15 (28.3)	
**Liver cirrhosis, n (%)**	Y	40 (74.1)	36 (67.9)	0.483
	N	14 (25.9)	17 (32.1)	
**Intrahepatic metastases, n(%)**	Y	7 (13.0)	13 (24.5)	0.125
	N	47 (87.0)	40 (75.5)	
**Distant metastases, n(%)**	Y	5 (9.3)	15 (28.3)	*0.012
	N	49 (90.7)	38 (71.7)	
**TNM stage, n (%)**	Ⅰ+Ⅱ	32 (59.3)	15 (28.3)	**0.0013
	Ⅲ+Ⅳ	22 (40.7)	38 (71.7)	
**Tumor differentiation, n (%)**	Well	4 (7.4)	3 (5.7)	*0.025
	Moderately	39 (72.2)	26 (49.0)	
	Poorly	11 (20.4)	24 (45.3)	
