# Supplementary material for: Activation of VIPR1 suppresses hepatocellular carcinoma progression by regulating arginine and pyrimidine metabolism
Source: Int J Biol Sci. 2022 Jul 4;18(11):4341–56. doi: 10.7150/ijbs.71134 (PMC9295067; doi:10.7150/ijbs.71134)
Supplement: Supplementary file 1 — Supplementary materials and methods, figures and tables. [file ijbsv18p4341s1.pdf]

Supporting Fig. S1A-D

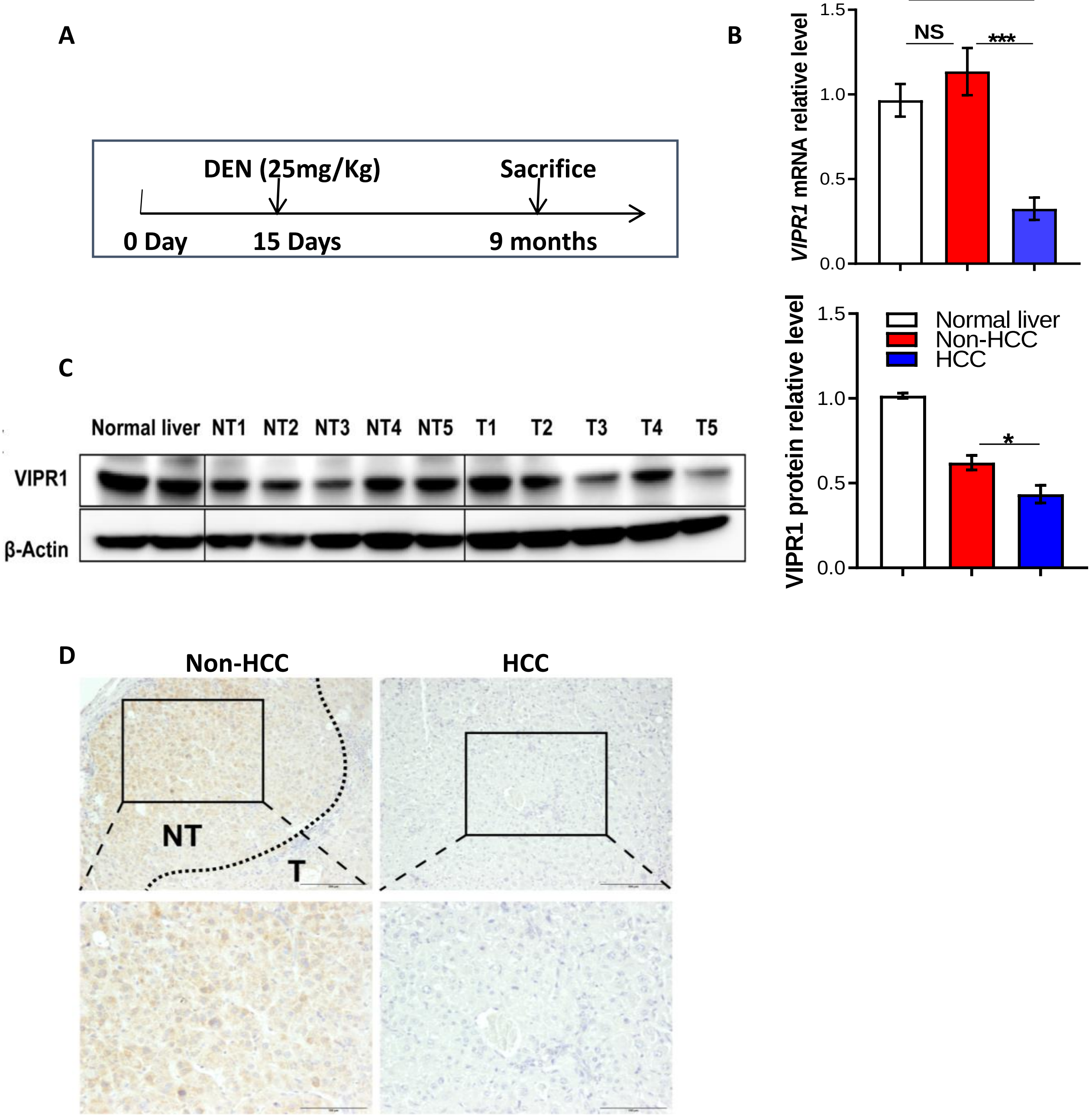

Supporting Fig. S1A-D. Hepatic VIPR1 expression is downregulated in mouse model of DEN-induced HCC. (A) Scheme for the establishment of Diethylnitrosamine (DEN)-induced liver cancer model. **(B)** *Vipr1* mRNA level measurement in Normal mouse liver (n=4), adjacent non-HCC tissues (n=8) and HCC tissues (n=8). **(C)** VIPR1 protein level determination of Normal mouse liver (n=2), adjacent non-HCC tissues (n=5) and HCC tissues (n=5). **(D)** Representative micrographs of immunohistochemistry staining of VIPR1 in Non-HCC and HCC regions in DEN-induced cancer model. Values represent means±SEM. **P*< 0.05, ***P*< 0.01, ****P*< 0.001.

Supporting Fig. S1E-H

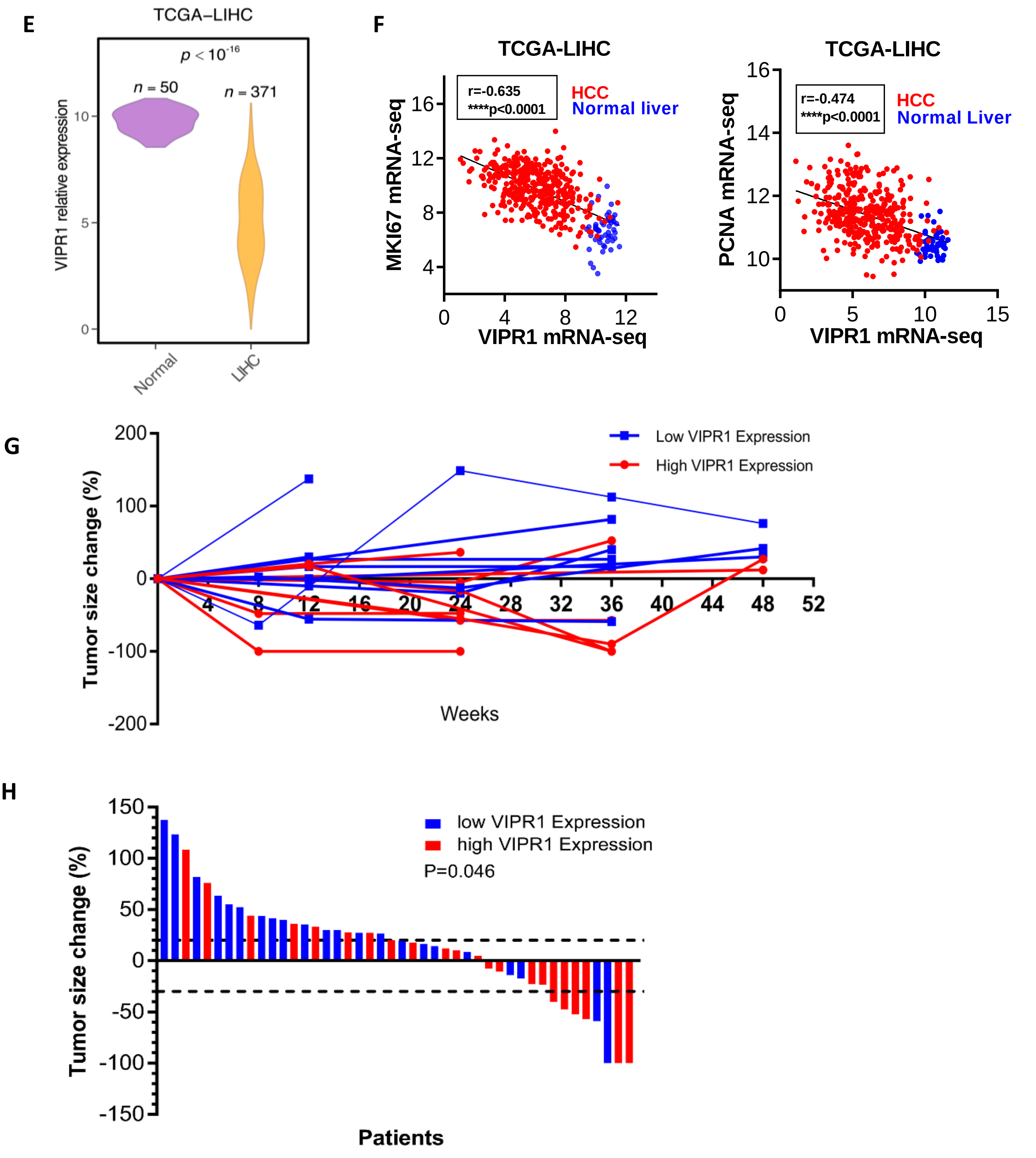

Supporting Fig. S1E-H. Role of VIPR1 expression in human HCC proliferation, tumor size changes. (E) *VIPR1* level comparison between normal liver (n=50) and HCC tissue (n=371) in TCGA-LIHC cohort. **(F)** Pearson's correlation between *VIPR1* and *MKI67* or *PCNA*. **(G)** Spider plot analysis was performed to evaluate recurrence in VIPR1^{high} (n=9) and VIPR1^{low} (n=10) subgroups in 1 year post surgery. The assessment was guided by RECIST version 1.1 criteria. **(H)** Waterfall plot of percentage change from baseline in size of target recurrent tumor lesion in VIPR1^{high} (n=22) and VIPR1^{low} (n=22) subgroups. Abbreviations: ROC: receiver operating characteristic; CI: Confidence Interval; AUC: area under curve. **P*< 0.05.

Supporting Fig. S2

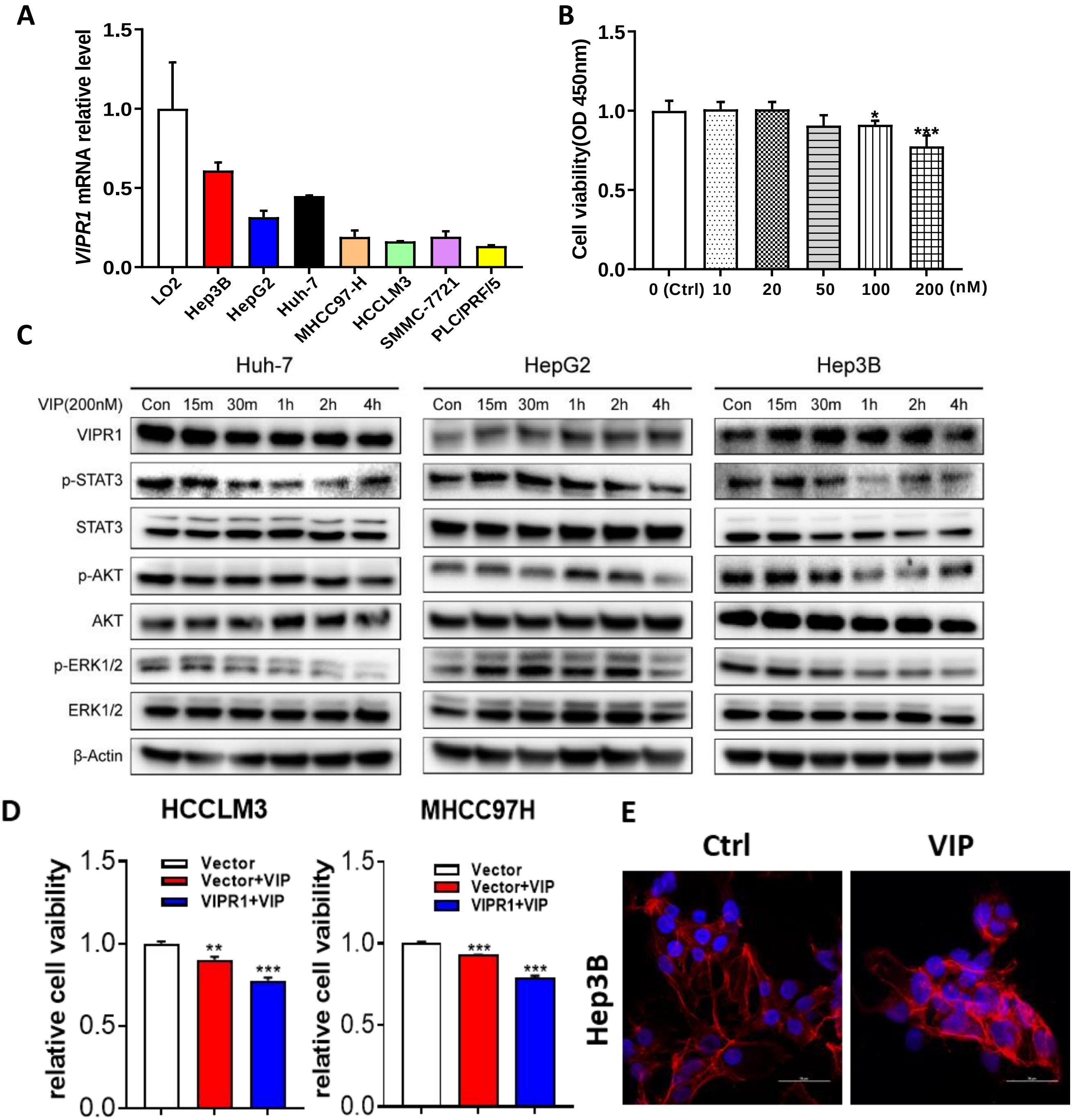

Supporting Fig. S2. Activation of VIP/VIPR1 signaling suppresses oncogenic signaling pathway and HCC growth. (A) *VIPR1* mRNA measurement in human hepatocyte cell line (LO2) and different human HCC cell lines. **(B)** Cell viability change after VIP treatment *in vitro* for 24 hours with different concentrations (0, 10nM, 20nM, 50nM, 100nM, 200nM). **(C)** Phosphorylation of STAT3, AKT, and ERK1/2 were partially inhibited after 200nM VIP treatment *in vitro*. **(D)** CCK8 assay for VIP/VIPR1 activated HCCLM3 and MHCC97H cells. **(E)** Representative micrographs of Phalloidin staining for cytoskeletal structure in Ctrl and VIP treated Hep3B cells. Blue: DAPI; Red: F-Actin. Values represent means±SEM. **P*< 0.05, ***P*< 0.01, ****P*< 0.001.

Supporting Fig. S3

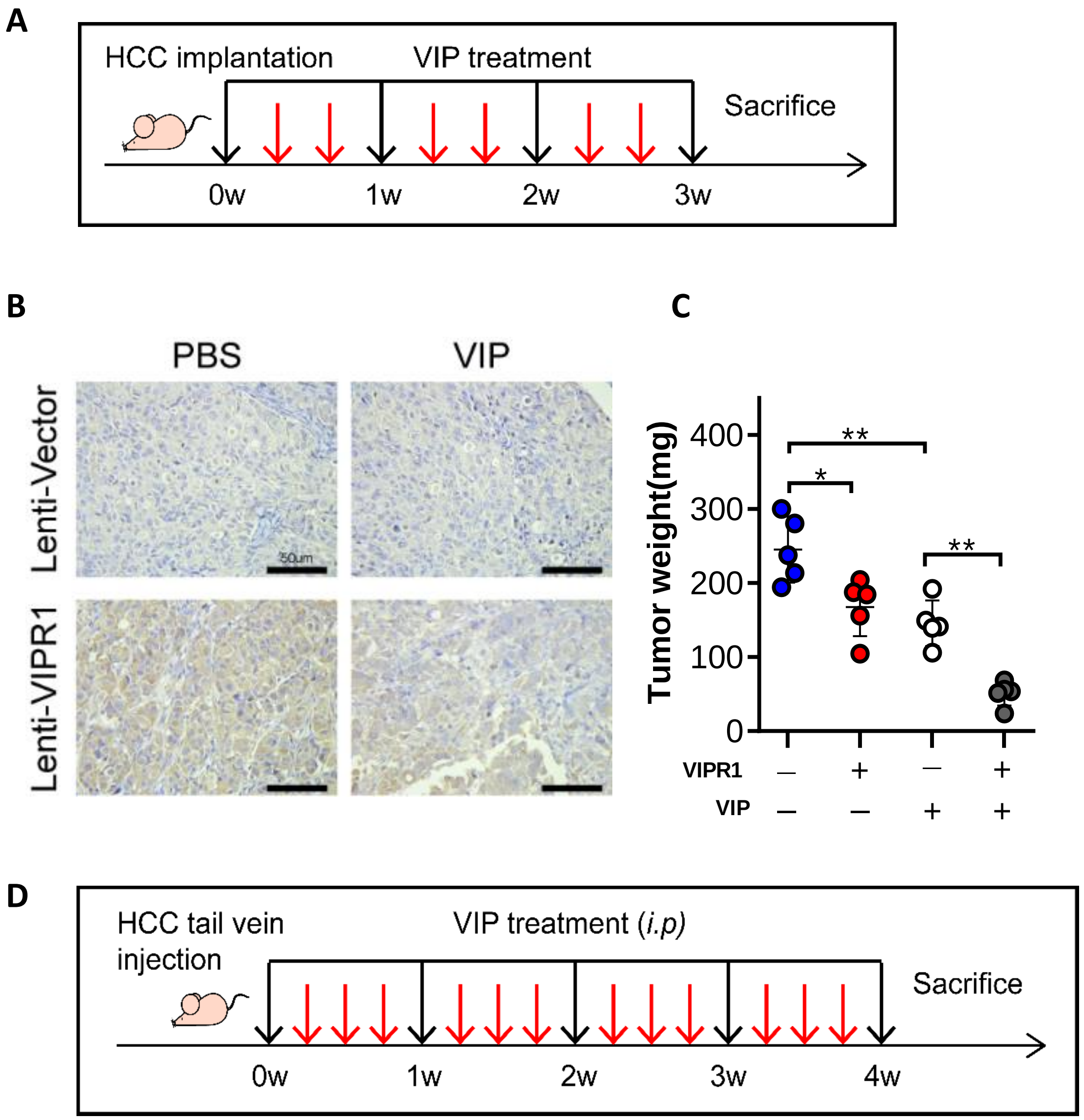

Supporting Fig. S3. VIP treatment inhibits HCC growth and metastasis *in vivo*. (A) The scheme shows that nude mice with HCC xenografts were treated with VIP twice per week (i.v., 300ug/kg). The mice were sacrificed 3 weeks later. **(B)** Representative micrographs of VIPR1 staining in HCCLM3-VIPR1 (overexpression group) and HCCLM3-Vector cells (Ctrl group). **(C)** Tumor weight (/mg) measurement in each group after sacrificing the mice. **(D)** Scheme for VIP *in vivo* treatment in metastatic model. In panels A and D, Red arrows mean VIP treatment, and black arrows indicate time points for sample collection. Values represent means±SEM. **P*< 0.05, ***P*< 0.01, ****P*< 0.001.

Supporting Fig. S4

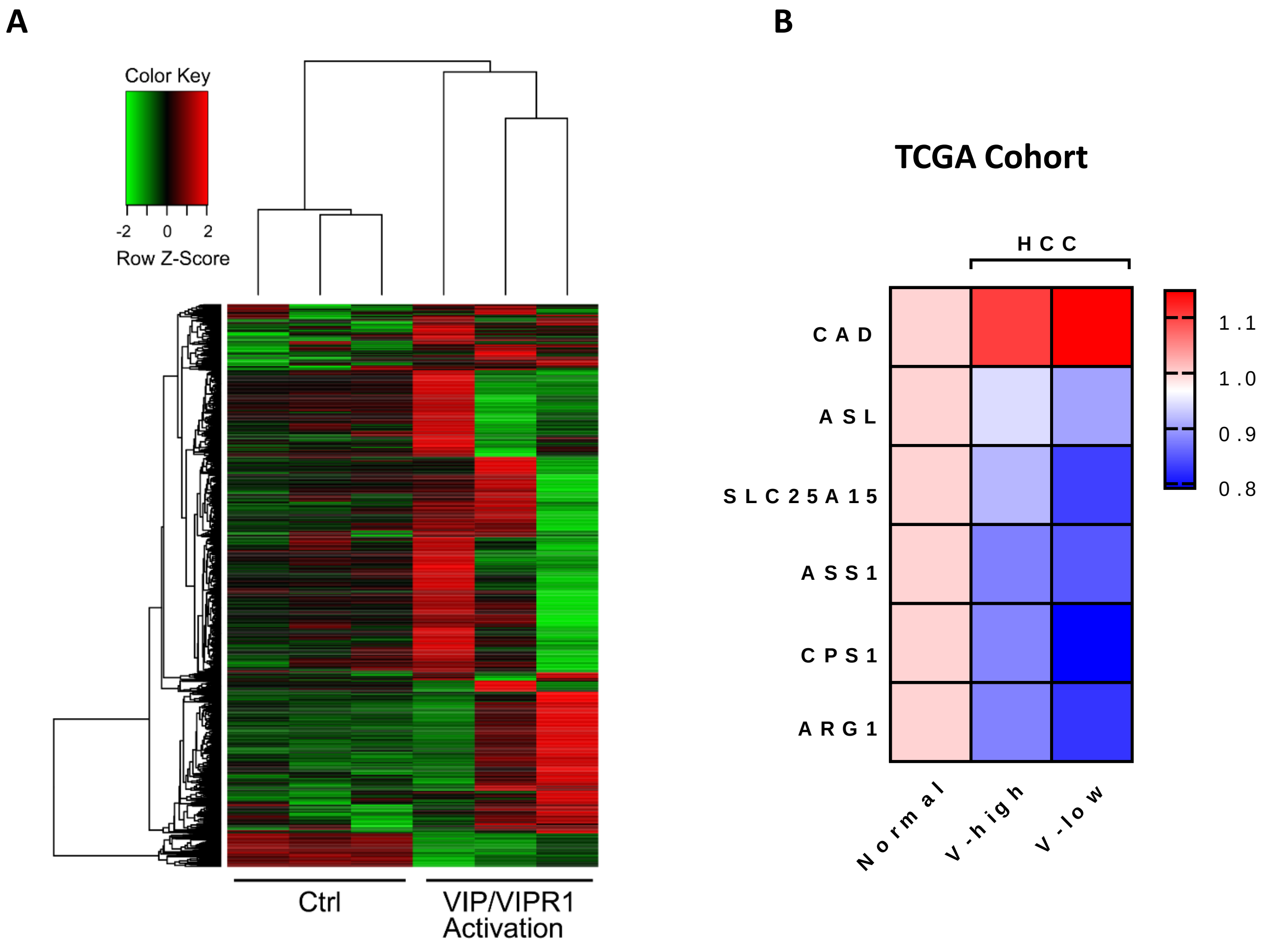

Supporting Fig. S4. Transcriptional signature correlates with VIP/VIPR1 signal activation. (A) Heatmap shows the alteration of transcriptional profiles after VIP/VIPR1 activation in Huh-7 cells. **(B)** Heatmap shows the difference of arginine metabolism-related genes expression in normal liver,VIPR1^{high} and VIPR1^{low} HCC of TCGA cohort.

Supporting Fig. S5

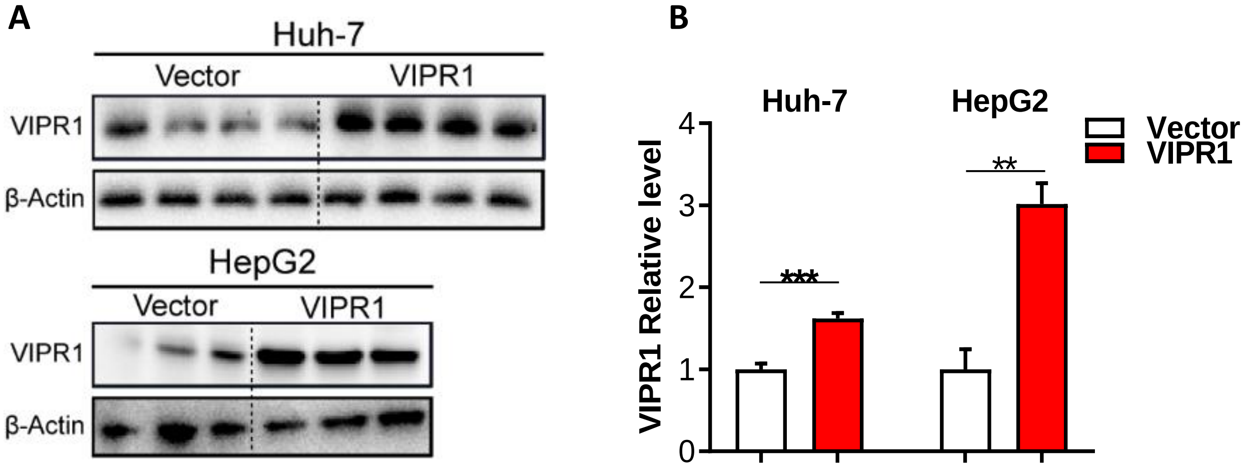

Supporting Fig. S5. Fold change of VIPR1 protein level after plasmid transfection in Huh-7 and HepG2 cells. (A). Protein level of VIPR1 were determined by performing western blot analysis. (B). Statistics of western blot results. Values represent means±SEM. ***P*< 0.01, ****P*< 0.001.

Supporting Fig. S6

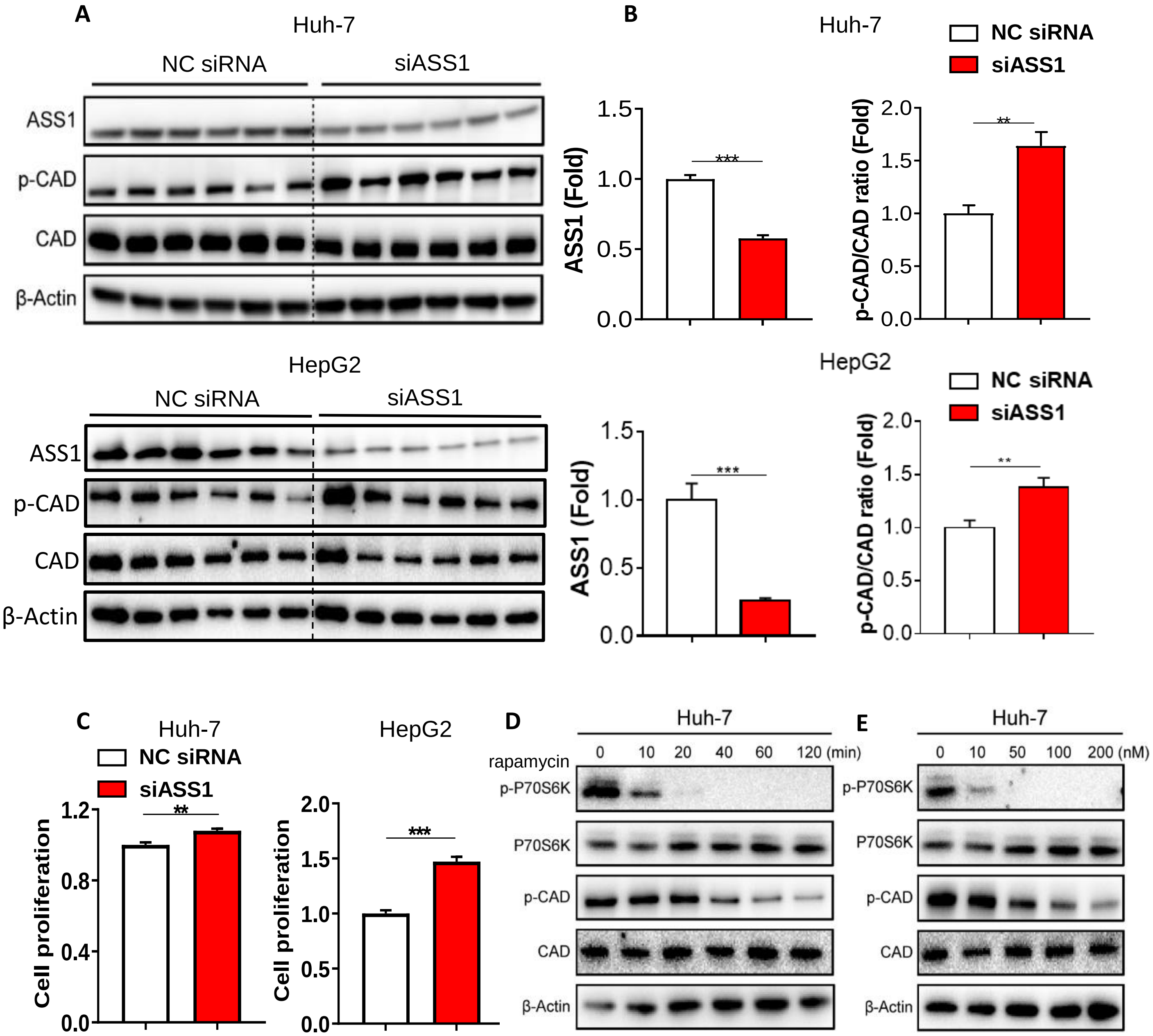

Supporting Fig. S6. ASS1 reduction promotes HCC cell proliferation and upregulates CAD phosphorylation via the activation of mTOR/p70S6K pathway. (A) Changes of ASS1 expression and p-CAD/CAD ratio in Huh-7 and HepG2 cells after ASS1 knockdown by siASS1 transfection. **(B)** Quantification of fold change for ASS1 and p-CAD/CAD ratio after siASS1 transfection in Huh-7 and HepG2 cells. **(C)** Fold change of cell proliferation in HepG2 cells (left Right panel) and Huh-7 cells (right Left panel) after siASS1 transfection. **(D, E).** Time- and dose-dependent manner of rapamycin treatment resulted decrease of CAD phosphorylation in Huh-7 cells (rapamycin concentration range: 0-200nM; duration: 0-120min). Values represent means±SEM. ***P*< 0.01, ****P*< 0.001.

Supporting Fig. S7

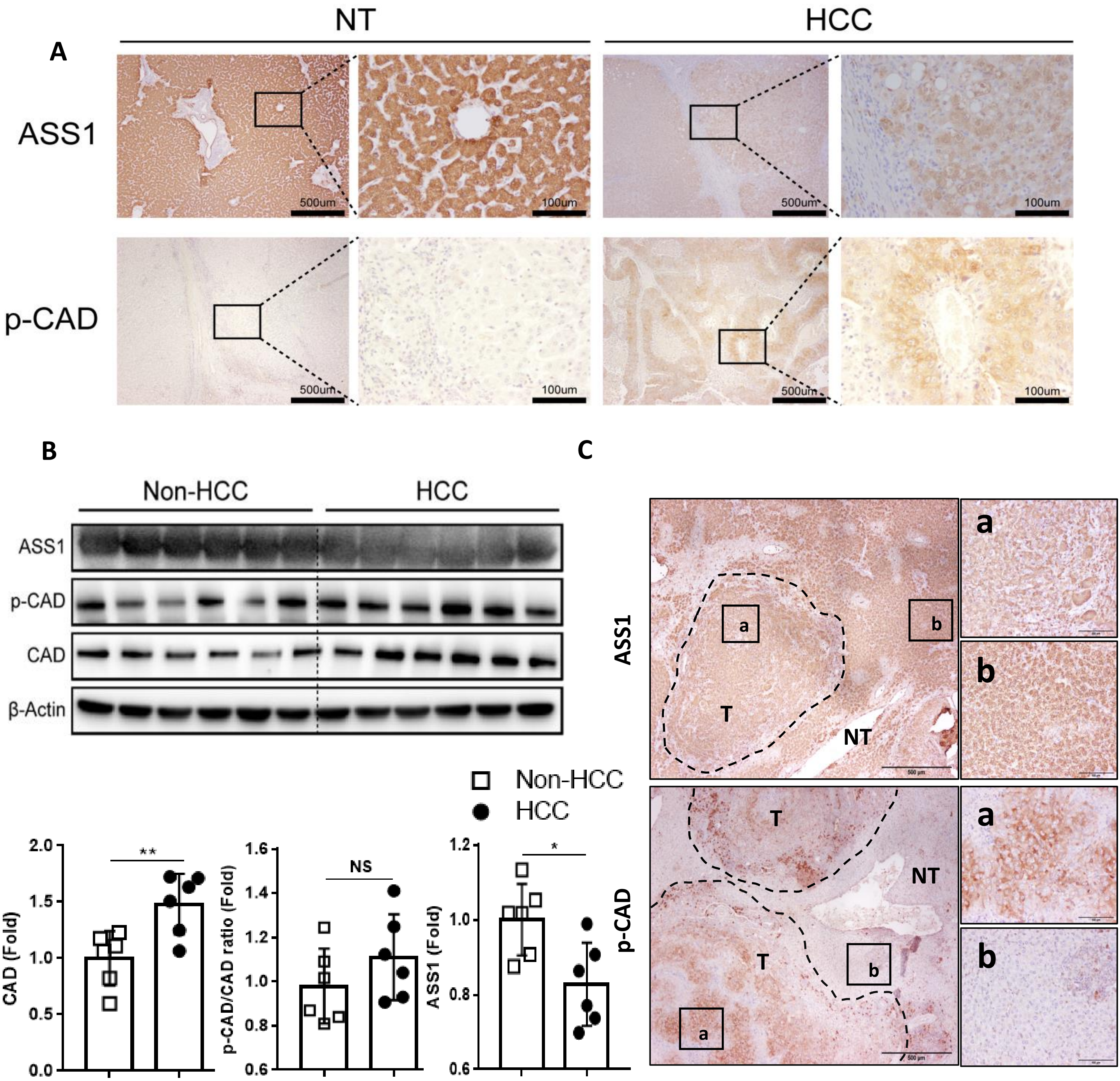

Supporting Fig. S7 (A): Representative IHC staining of ASS1 and p-CAD in human non-tumor liver and HCC tissue sections. (B, C) ASS1 and phosphorylated CAD (p-CAD) expression in DEN-induced HCC model. Protein levels of ASS1, total CAD and p-CAD in HCC tissues (n=6) and non-HCC liver tissues (n=6). Representative images of ASS1 and p-CAD in mouse HCC sections. The specific areas in tumor region (labeled 'T') surrounded by dotted line and non-tumorous region (labeled 'NT') are enlarged in 'a' and 'b'. Values represent means±SEM. **P*< 0.05, ***P*< 0.01.

Supplementary Materials and Methods

Human Cohort Study

A 107-patient cohort diagnosed with hepatocellular carcinoma (HCC) was enrolled in our study for investigating the significance of VIPR1 in HCC clinical prognosis. All HCC patients received surgical resection in Xiangya Hospital during 2005-2019. Human samples were obtained under informed consent. The clinicopathological characteristics, including age, gender, pathology, tumor size, tumor multiplicity, cirrhosis, vascular invasion and differentiational status, were available for all patients. Diagnosis of HCC and non-tumor adjacent liver tissues were confirmed based on histological findings by independent pathologists. The assessment of HCC tumor burden after surgery and treatment was guided by RECIST version 1.1 Criteria. Frozen specimens at -80°C of non-tumor adjacent liver tissue (liver tissue close to the tumorous region) and HCC tissues were used to extract total RNA and protein for analyses. Serum samples of HCC patients were collected before surgery and were used to measure urea concentration by using the Urea Assay kit (Abcam, ab83362). Paraffin-embedded surgical tissues (non-tumor liver and HCC tissues) were used for immunohistochemistry analyses. The information for all HCC cohorts enrolled in this study are listed in **Table S1-Table S6**.

RNA isolation and quantitative reverse transcription PCR (RT-qPCR)

Total RNA was extracted from liver tissues and cell samples using TRIzol reagent (Invitrogen, Carlsbad, CA) following the manufacturer's instruction. One microgram (1μg) RNA was reverse-transcribed into cDNA by using High Capacity cDNA Reverse Transcription kit (Thermo Fisher Scientific). RT-qPCR was performed by SYBR Green Realtime PCR master mix. The mRNA levels were measured by QuantStudio^{TM} 6 Real-Time PCR System (278861830; Thermo Fisher Scientific). The expression levels of target genes were normalized to GAPDH (for human genes) and 18S rRNA (For mouse genes) expression. Comparative Ct (2-ΔΔCt) method was performed to quantify the mRNA expression level. All primers used for RT-qPCR are listed in **Table S7**.

Western Blotting

Liver tissues or cell lysates were homogenized in RIPA lysis buffer containing cocktail of protease inhibitors (Santa Cruz, CA) according to the manufacturer's instruction at 4°C and centrifuged at 10,000 g for 10 minutes. Protein extracts were quantified using BCA Protein Assay Kit (Thermo Fisher, Waltham, MA, USA), mixed with loading buffer, and then were subjected to 4-12% Bis-Tris protein gels (Bio-Rad, Hercules, CA, USA) and transferred to nitrocellulose membranes (Thermo

Fisher, Waltham, MA, USA). Protein bands were visualized with SuperSignal West Femto Maximum Sensitivity Substrate (Thermo Fisher, Waltham, MA, USA). Primary antibodies against proteins of interest are listed in **Table S8**. Secondary horseradish peroxidase-conjugated antibodies (Santa Cruz Biotechnology, Dallas, TX, USA) were used.

Cell culture and *in vitro* treatment

Human hepatocyte cell line LO2 and human hepatocellular carcinoma cell lines (including: MHCC97H, HCCLM3, SMMC-7721, PLC/PRF/5) were purchased from The American Type Culture Collection (ATCC) and Biotech company (Zhong Qiao Xin Zhou Biotechnology Co.,Ltd., Shanghai, China). Another 3 human hepatocellular carcinoma cell lines, including HepG2, Hep3B, Huh-7, were kindly provided by Dr. Hongbing Wang, University of Maryland. Hep3B cells were cultured in a mixture of Minimum Essential Medium, 10% fetal bovine serum and penicillin-streptomycin. Other cell lines were cultured in the complete medium composed of Dulbecco's modified Eagle's medium, 10% fetal bovine serum and penicillin-streptomycin (all from Thermo Fisher Scientific, Waltham, MA, USA). Cells underwent short-term starvation (2hrs) were *in vitro* treated with VIP (human, rat, mouse, rabbit, canine, porcine) (1911/1; Tocris) at a final concentration of 200nM in routine culture conditions. In mechanistic study, Rapamycin (PHZ1235, Thermo Fisher Scientific) diluted in DMSO were used to treat cells at the specific concentration (0nM, 10nM, 50nM, 100nM, 200nM were used).

Cell counting kit-8 (CCK-8) assay

In vitro cell proliferation and viability were determined by colorimetric method by using Cell counting kit-8 (CCK-8) assay (Cat # DJDB4000X, VitaScientific) according to the manufacturer's instruction. Cell viability was presented as absorbance value at 450nm. Cell proliferation was monitored at 4 different timepoints during consecutive 3 days. Similar method was previously described in [1].

Colony formation assay and Wound healing assay

These two assays were used to determine HCC cell proliferation and migration potential *in vitro*, which were performed as described previously [1, 2]. All experiments were performed in triplicate. The MHCC97H and HCCLM3 cells were seeded in 6-well plates and were cultured in the routine condition. 200 uL tips were used for the wound healing when the cell confluency was above 90%. Then the cells were washed with PBS and cultured with a low concentration of serum. Photos were taken at two timepoints (0h, 24h) and statistically analyzed by using ImageJ software (National Institutes of Health, Bethesda, MD).

Plasmid and siRNA transfection

VIPR1 was a gift from Gavin Wright (Addgene plasmid # 51865; http://n2t.net/addgene:51865; RRID:Addgene_51865). Transient plasmid transfection was performed with Lipofectamine 3000 reagent according to the manufacturer's instruction (Invitrogen, USA). siRNA targeting ASS1 (siASS1) (P/N: AM51331; Lot# ASO2GXJ2 ; siRNA ID#:117838) and its negative control siRNA (Silencer™ Negative Control No. 2 siRNA; Catalog#: AM4613) were purchased from Thermo Fisher Scientific. siRNA transfection was performed with Lipofectamine RNAiMAX transfection reagent (Invitrogen, USA) following the manufacturer's instruction. The transfection efficiency was confirmed by performing Western Blotting.

Lentivirus infection

The lentiviral vector GV358 (Ubi-MCS-3FLAG-SV40-EGFP-IRES-Puromycin) containing human VIPR1 (NM_004624) and its Control vector, and lentiviral vector GV260 (Ubi-MCS-firefly_Luciferase-IRES-Puromycin) for bioluminescent imaging analysis were constructed from Shanghai GeneChem, China. Cell infection was performed according to the manufacturer's instruction. To select stably transduced cell clones, the infected cells were cultured with puromycin (2.5ug/mL) for 2 weeks; RT-qPCR was then performed to determine the overall infection efficiency.

Urea concentration measurement

Urea Assay Kit (ab83362, Abcam) was used to measure the urea concentration in HCC serum samples and cell culture medium according to the manufacturer's instruction. The samples should be diluted by 1xPBS to a proper concentration range for analysis. The standards added to 96-well plates were used for plotting standard curve. Urea in samples was acted on by enzymes to form a product; the reaction mixture was incubated at 37°C for 1h, and measured the absorbance by using microplate reader (ODmax=570nm). Absorbance is directly proportional to the urea concentrationin the system.

Immunohistochemistry (IHC) and hematoxylin and eosin (H&E) staining

Paraffin-embedded tissues were used for immunohistochemistry staining. 4-μm-thick paraffin sections were stained with hematoxylin and eosin (H&E) for histological analysis. For IHC staining, heat-induced epitope retrieval was carried in 3% citrate buffer, endogenous peroxidases were blocked with 3% H2O2 for 20 minutes, and non-specific binding was blocked by using 3% normal goat serum for 1 hour at room temperature. Slides were then incubated with primary antibody overnight at 4°C. After PBS washing, slides were subsequently incubated with anti-mouse or anti-rabbit secondary antibodies (SignalStain® Boost IHC Detection Reagent, Cell Signaling Technology, Danvers, MA, USA) for 1 hour at room temperature. Staining results were developed and visualized by Vectastain Elite ABC Staining Kit DAB peroxidase substrate kit (Vector Laboratories, Inc., Burlingame, CA) according to the

manufacturer's instruction, and then slides were counterstained with hematoxylin. The analysis of positive area was determined with ImageJ software (National Institutes of Health, Bethesda, MD).

Immunofluorescent staining for adherent cells

Cell adherent sections were fixed in 4% paraformaldehyde (4% PFA, pH7.4) for 10 minutes and washed with PBS three times. Slides were blocked in blocking solution (3% bovine serum albumin in PBS) for 1 hour at room temperature and incubated with primary antibodies (listed in **Table S8**) overnight at 4°C. After PBS washing, slides were incubated with the fluorescence conjugated antibodies (listed in **Table S8**) for 1 hour at room temperature. Nuclear staining was obtained by incubation with 1 mg/mL 4', 6'-diamino-2-phenylindole (DAPI) for 5 min at room temperature. Images were acquired using LSM 710 confocal microscope (Zeiss, Thornwood, NY, USA).The images were analyzed by the con-focal microscopic system from Zeiss (Thornwood, NY).

HCC tumor cell derived xenograft (CDX) model

The method for building HCC cell derived xenograft (CDX) model was discussed in Methods and materials and figure legends of the main text. Tumor size was measured every 3 days. Tumor volume was calculated by using the following formula: volume $=1/2\times(\text{length}\times\text{width}^2)$. All mice were sacrificed at day 21. Tumor tissues were isolated and embedded in paraffin for Ki-67 (AY0064, ABZOOM) staining and analysis.

***In vivo* bioluminescent imaging on metastatic model**

Bioluminescence imaging (BLI) method was utilized to visualize and quantify bioluminescent signal of metastatic HCC in mouse model (OD-Prkdcem26Cd52Il2rgem26Cd22/Nju (NOD/SCID IL2rg$^{-/-}$ mice, or NCG mice; Nanjing Biomedical Research Institute of Nanjing University). Luciferase Vector GV260 (Ubi-MCS-firefly_Luciferase-IRES-Puromycin) labeled HCCLM3 cells was injected via tail vein (More details are mentioned in main text). The mice were anaesthetized under 2% isoflurane gas, then were intraperitoneally injected with D-Luciferin (Sodium Salt D 40901ES01, YEASEN, China), and were imaged 10-15 minutes after the injection in an IVIS imaging system (IVIS® In Vivo Imaging Systems, Spectrum BL; PerkinElmer, MA) according to the manufacturer's instruction under anesthesia. BLI signal is quantified in regions of interest (ROIs). Images were analyzed by using Living Image 4.3.1 software (PerkinElmer, MA). Metastatic lesions were measured once a week after model establishment, over the next 4 weeks. After sacrificing the mice, spleen, liver and lung tissues were isolated and measured to quantify the metastases. The targeted organs were collected and fixed in formalin, and embedded in paraffin for H&E staining.

References

[1]. Niu W, Luo Y, Zhou Y, Li M, Wu C, Duan Y, et al. BRD7 suppresses invasion and metastasis in breast cancer by negatively regulating YB1-induced epithelial-mesenchymal transition. J Exp Clin Cancer Res. 2020;39(1):30.

[2]. Xue Z, Zhao J, Niu L, An G, Guo Y, Ni L. Up-Regulation of MiR-300 Promotes Proliferation and Invasion of Osteosarcoma by Targeting BRD7. PLoS One. 2015;10(5):e0127682.

Table S1. Clinical information and *VIPR1* level of HCC patients for waterfall plot analysis

Patient No.	Gender	Age	Etiology	*VIPR1* relative expression	Change of tumor size (%)
1	M	54	HBV	0.004	-58.9
2	M	53	HBV	0.006	55.2
3	M	47	HBV	0.006	-100.0
4	M	40	HBV	0.009	40.0
5	M	69	HBV	0.01	27.3
6	F	45	HBV	0.01	-13.8
7	F	53	HBV	0.01	41.7
8	M	49	HBV	0.01	16.7
9	M	61	HBV	0.016	63.6
10	F	55	HBV	0.018	-17.4
11	M	38	HBV	0.018	30.0
12	M	67	HBV	0.019	52.4
13	M	39	HBV	0.021	43.8
14	M	41	HBV	0.024	30.0
15	F	38	HBV	0.025	81.8
16	M	30	HBV	0.026	8.7
17	M	49	HBV	0.026	26.7
18	M	34	HBV	0.03	137.5
19	F	54	HBV	0.031	35.7
20	M	52	HBV	0.031	123.3
21	M	53	HBV	0.037	14.3
22	M	52	HBV	0.045	19.0
23	M	47	HBV	0.047	-7.7
24	M	59	HBV	0.047	20.0

25	M	64	HBV	0.057	-10.7
26	M	75	HBV	0.067	44.1
27	M	58	HCV	0.071	33.3
28	M	63	HBV	0.077	-57.1
29	M	46	HBV	0.079	36.4
30	M	53	HBV	0.08	12.2
31	M	51	HBV	0.083	-100.0
32	M	59	HBV	0.127	108.3
33	M	49	HBV	0.143	-23.0
34	M	62	HBV	0.162	5.0
35	M	52	HBV	0.216	17.6
36	F	73	Others	0.227	-23.5
37	M	50	HBV	0.285	-100
38	M	65	HBV	0.393	27.5
39	M	43	HCV; Alcohol	0.473	-52.2
40	M	32	HBV	0.484	76.2
41	M	55	HBV	1	27.3
42	M	58	HBV	2.068	-40.0
43	M	51	Others	2.777	10.4
44	M	48	HBV	3.659	-47.5

Table S2. Clinical parameters of HCC patients for RT-qPCR analysis of Urea cycle related genes

Patient No.	Gender	Age	Etiology	AFP (ng/mL)
1	M	58	HCV	6.09
2	F	54	HBV	4.31
3	M	54	HBV	645.80
4	M	69	HBV	736.45
5	M	65	HBV	6.10
6	M	59	HBV	7.28
7	M	30	HBV	8.28
8	F	45	HBV	10890.00
9	M	67	HBV	29.60
10	M	52	HBV	109.60
11	M	64	HBV	4.77
12	M	61	HBV	30.82
13	M	34	HBV	1.29
14	M	39	HBV	800.00
15	M	56	Others	8.34
16	M	52	Others	1210.00
17	F	61	Others	4.08
18	M	52	HBV	2.21
19	M	75	Others	3.71
20	M	53	HBV	5.67
21	M	46	HBV	5.27
22	M	48	HBV	1210.00
23	M	43	HCV; Alcohol	1.94
24	F	40	HBV	759.80

Table S3. Clinical information of HCC patients for urea concentration test

Variables		High VIPR1 Group (n=17)	Low VIPR1 Group (n=18)	P value
Age (years)		50.59 ± 3.151	52.83 ± 2.041	0.5494
Gender, n (%)	Male	15 (88.2)	15 (83.3)	>0.9999
	Female	2 (11.8)	3 (16.7)	
AFP, n (%)	≥100ng/ml	10 (58.8)	12 (66.7)	0.7332
	<100ng/ml	7 (41.2)	6 (33.3)	
Child-Pugh classification	A	14 (82.4)	14 (77.8)	>0.9999
	B+C	3 (17.6)	4 (22.2)	
Liver cirrhosis, n (%)	Y	8 (47.1)	14 (77.8)	0.0858
	N	9 (52.9)	4 (22.2)	
Tumor differentiation, n (%)	Well	6 (35.3)	2 (11.1)	0.1661
	Moderately	11 (64.7)	15 (83.3)	
	Poorly	0 (0)	1 (5.6)	

Table S4. Clinical data of HCC patients for ROC curve analysis

Patient No.	Gender	Age	Etiology	VIPR1 relative expression	AFP (ng/mL)	Urea (mmol/L)	Relapse
1	M	49	HBV	0.143	244.89	3.75	Y
2	M	65	HBV	0.393	6.1	5.50	N
3	F	45	HBV	0.01	10890	3.48	Y
4	M	52	HBV	0.031	109.6	6.70	Y
5	M	53	HBV	0.006	137.3	2.20	Y
6	M	39	HBV	0.021	800	4.78	Y
7	M	52	HBV	0.045	2.21	4.16	N
8	M	43	HCV, Alcohol	0.473	1.94	3.76	N
9	M	59	HBV	0.047	12.83	4.59	N
10	F	50	Others	0.143	3.49	2.71	Y
11	M	32	HBV	0.484	499.23	3.19	N
12	M	52	HBV	0.231	7.69	6.01	Y
13	M	46	HBV	0.079	1210	7.15	Y
14	M	63	HBV	0.077	5.32	3.32	Y
15	M	62	Alcohol	0.294	1210	6.13	N
16	F	37	HBV	0.041	1210	2.52	Y
17	M	42	HBV	0.086	1.13	5.13	N
18	F	56	HBV	0.143	1210	2.93	Y
19	M	41	HBV	0.024	1.24	4.01	Y
20	M	60	Alcohol	0.417	1.92	3.97	N
21	M	51	HBV	0.092	800	4.96	N
22	M	57	HBV	0.146	4.55	5.00	N
23	M	53	HBV， Alcohol	0.007	108.6	6.40	Y
24	M	67	HBV	0.115	4.27	8.21	N
25	F	53	HBV	0.08	1210	3.05	Y
26	M	70	HBV	0.521	15.88	3.72	N
27	M	51	HBV	0.083	242.24	4.45	Y
28	M	50	HBV	0.14	7.41	4.00	N
29	M	56	HBV	0.076	5.26	3.37	Y
30	M	42	HBV	0.15	547.61	3.49	N
31	M	77	HBV	0.012	2.45	5.44	N
32	M	45	HBV， Alcohol	0.016	191.15	2.10	Y

33	F	51	HBV	0.079	1210	4.78	Y
34	M	47	HBV， Alcohol	0.014	1210	3.51	Y
35	M	56	HBV	0.025	1.89	5.34	N
36	F	62	Others	0.014	9.23	2.34	Y
37	M	49	HBV	0.527	8.32	2.80	Y
38	M	39	HBV	0.037	223.8	5.96	Y
39	M	26	HBV	0.022	245.161	4.63	Y
40	F	69	HBV	0.647	800	4.33	N
41	M	38	HBV	0.186	1210	3.25	Y
42	M	45	HBV	0.033	300	2.15	Y
43	M	50	HBV， Alcohol	0.571	1210	2.01	Y

Table S5. Clinical information of HCC patients for p-CAD IHC staining analysis

Patient No.	Age	Gender	Etiology	p-CAD Score	Relapse
1	58	M	HCV	++	0
2	54	F	HBV	+	0
3	65	M	HBV	+	0
4	53	M	HBV	+++	0
5	30	M	HBV	+++	0
6	64	M	HBV	-	0
7	53	F	HBV	+	0
8	52	M	HBV	-	0
9	75	M	HBV	+	0
10	43	M	HBV, Alcohol	++	0
11	59	M	HBV	++	0
12	51	M	HBV	+	0
13	49	M	HBV	+	0
C	71	M	HCV	-	0
D	66	M	HBV, Alcohol	+	0
E	59	M	HCV	+	0
17	55	M	HBV	+	1
18	48	M	HBV	+	1
19	54	M	HBV	+++	1
20	45	F	HBV	-	1
21	67	M	HBV	+	1
22	52	M	HBV	+	1
23	34	M	HBV	+++	1
24	39	M	HBV	++	1
25	56	M	Others	++	1
26	52	M	Others	+++	1
27	61	F	Others	+++	1
28	46	M	HBV	++	1
29	48	M	HBV	+++	1
30	73	F	Others	++	1
31	40	F	HBV	++	1
32	39	M	HBV	++	1
33	55	M	Others	+	1
34	47	M	HBV	+++	1
A	48	M	HCV	+++	1
B	56	M	HCV	++	1

Table S6. Clinical information of Spider plot analysis

Patient No.	Age	Gender	tumor size change (%)					
			8w	12w	24w	36w	48w	VIPR1 expression
1	55	M			-55	-90	27.3	High
2	54	M		-55.4		-58.9		Low
3	50	M		17.6		-100		High
4	32	M	-64		148.7	112.4	76.2	Low
5	46	M		20.5	36.4			High
6	38	F				81.8		Low
7	63	M			-51.7	-51.7		High
8	49	M	-47.7		-47.5			High
9	34	M		137.5				Low
10	41	M		0			30	Low
11	38	M		30				Low
12	53	M	2.4		-12.5		41.7	Low
13	51	M		0	-16.3	-100		High
14	40	M		-10	-20	40		Low
15	53	F					12.2	High
16	47	M	-100		-100			High
17	49	M		16.7		16.7		Low
18	26	M		26.7	26.7			Low
19	48	M			-4.8	52.4		High

Table S7. Primers used for RT-qPCR analysis

Gene	Forward primer (5′–3′)	Reverse primer (5′–3′)
VIPR1(H)	CCCCTGGGTCAGTCTGGTG	GAGACCTAGCATTCGCTGGTG
GAPDH(H)	GACCTGACCTGCCGTCTAGAAA	CCTGCTTCACCACCTTCTTGA
Vipr1(M)	GATGTGGGACAACCTCACCTG	TAGCCGTGAATGGGGGAAAAC
18s(M)	AACTTTCGATGGTAGTCGCCGT	TCCTTGGATGTGGTAGCCGTTT
ASS1(H)	CTTGGGGCCAAAAAGGTGTTC	GAGGTAGCGGTCCTCATACAG
ASL(H)	CAGTGGACCCCATCATGGAGA	GGCTTTGCTGCCTTGAACATC
CPS1(H)	AGGCCCATGCCACAAATCATCA	GCCTGATGCCAGGTCTTGAA
OTC(H)	CGGCCCGTGTATTGTCTAGC	TAGCCAGGGTGTCCAAATCTG
SLC25A15(H)	CCTGAAGACTTACTCCCAGGT	GCGATGTTGGCGATTAGTGC
SLC25A13(H)	TGGACTGTATAGAGGTCTGTTGC	CCCTCACAAAATCGTTCACTGT

Notes: 'H' means human, 'M' means mouse

Table S8. Information of reagents used in the project

Reagent	Cat No.	Manufacturer
VIPR1 (used for IHC)	14878-1-AP	Proteintech
VPAC1 (used for WB)	AB2265	Millipore
Ki-67	AY0064	ABZOOM
	9027	Cell Signaling Technology
Cyclin E	20808	Cell Signaling Technology
Cyclin D1	2922	Cell Signaling Technology
β-Actin	ab8227	Abcam
Phospho-CAD (Ser1859)	67235	Cell Signaling Technology
CAD	93925	Cell Signaling Technology
ASS1	70720	Cell Signaling Technology
P70S6K	2708	Cell Signaling Technology
p-P70S6K(Thr389)	9234	Cell Signaling Technology
Stat3	12640	Cell Signaling Technology
Phospho-Stat3（Tyr705）	4113	Cell Signaling Technology
Akt	9272	Cell Signaling Technology
Phospho-Akt（Ser473）	4060	Cell Signaling Technology
p44/42 MAPK	4695	Cell Signaling Technology
Phospho-p44/42 MAPK	4376	Cell Signaling Technology
Urea Assay Kit	ab83362	Abcam
Secondary antibodies for IF staining	4409	Cell Signaling Technology
	4412	Cell Signaling Technology
Cell Counting Kit-8 (CCK-8)	DJDB4000X	VitaScientific
D-Luciferin, Sodium Salt	40901ES01	Yeasen
Avertin	M2910	Easycheck
VIP (human, rat, mouse, rabbit, canine, porcine)	1911/1	Tocris
